# Hypoxia-responsive nanoparticles for fluorescence diagnosis and therapy of cancer

**DOI:** 10.7150/thno.104190

**Published:** 2025-01-01

**Authors:** Yubing Zhang, Jiaqi Xing, Juan Jiang, Maoliang Liao, Guojun Pan, Yanfeng Wang

**Affiliations:** 1 State Key Laboratory of Advanced Drug Delivery and Release Systems, Key Laboratory for Biotechnology Drugs of National Health Commission, Key Laboratory of Rare and Rare Diseases in Shandong Province, School of Pharmacy (Institute of Pharmacy) of Shandong First Medical University, Jinan, Shandong 250117, China.; 2 School of Life Sciences, Shandong First Medical University and Shandong Academy of Medical Sciences, Jinan, Shandong, China.; 3 Shandong Luye Pharmaceutical Co., Ltd., China.; 4 Anhui Engineering Laboratory for Conservation and Sustainable Utilization of Traditional Chinese Medicine Resources, West Anhui University, Lu'an, 237012, Anhui, China.

**Keywords:** hypoxia, nanoparticles, prodrugs, tumor therapy, fluorescence imaging

## Abstract

Hypoxia, caused by rapid tumor growth and insufficient oxygen supply, is a defining characteristic of numerous solid tumors and exerts a significant influence on tumor growth, metastasis, and invasion. Early diagnosis and effective killing of tumor cells are crucial for cancer treatment. In recent years, the emergence of nanomaterials has overcome the difficulties in the delivery of chemotherapeutic drugs and contrast agents to tumor area. In this review, we summarize the development of hypoxia-responsive nanoparticles for fluorescence imaging and tumor therapy in the last five years, and further discuss their design strategies and applications in bioimaging. In addition, we discuss the therapeutic strategies of hypoxia-responsive prodrugs on different nanoplatforms and the future prospects of hypoxia-responsive nanomedicine in tumor therapy.

## 1. Introduction

The treatment of cancer consumes huge medical resources, and the high incidence and mortality of cancer also bring heavy burden on society. For example, the incidence and mortality rates of cancer were reported at 18.1 million cases and 9.6 million deaths in 2018, escalating to 20 million cases and 9.7 million deaths by 2022. The United States, for example, spends 124.6 billion US dollars annually on cancer 1, 2. Therefore, cancer has emerged as a global problem 3. Currently, cancer is usually treated using traditional methods such as chemotherapy, radiotherapy, and surgery. A variety of drugs have been developed to treat cancer in the early stage, but these small molecule chemotherapeutic drugs exhibit limited selectivity for tumors. This lack of selectivity can also induce apoptosis in healthy tissues, leading to more severe side effects 4, 5, such as bone marrow suppression, stomach pain, a significant drop in blood pressure, and reduced lymphocyte counts. Therefore, it is imperative to find drugs that can specifically target tumors.

When normal cells are stimulated to transform into tumor cells, the intracellular microenvironment changes, mainly manifested by decreased pH, elevated levels of reactive oxygen species (ROS), and the overexpression of certain enzymes 6-8. In addition, as tumor volume expands rapidly, the vascular structure becomes distorted and abnormal. These structural alterations impede the effective transport of nutrients, hinder the timely removal of metabolic waste, and restrict oxygen delivery, leading to the formation of hypoxic regions within the tumor 9-11. Therefore, hypoxia arises from a combination of the rapid proliferation of tumor cells and an inadequate oxygen supply. Since hypoxia is rarely observed in normal tissues, a variety of hypoxia-activated prodrugs have been developed by introducing hypoxia-responsive groups 12-15. These prodrugs remain non-toxic under normoxic conditions but can be activated to release the active drug under hypoxic environments 16, thereby significantly reducing the side effects of chemotherapeutic agents on normal cells during cancer therapy. Despite the substantial progress made in the development of hypoxia-activated prodrugs, some drugs still face challenges related to low solubility and low delivery efficiency. These challenges can be effectively addressed by integrating carefully designed nanomaterials with prodrugs.

**Figure 1:**
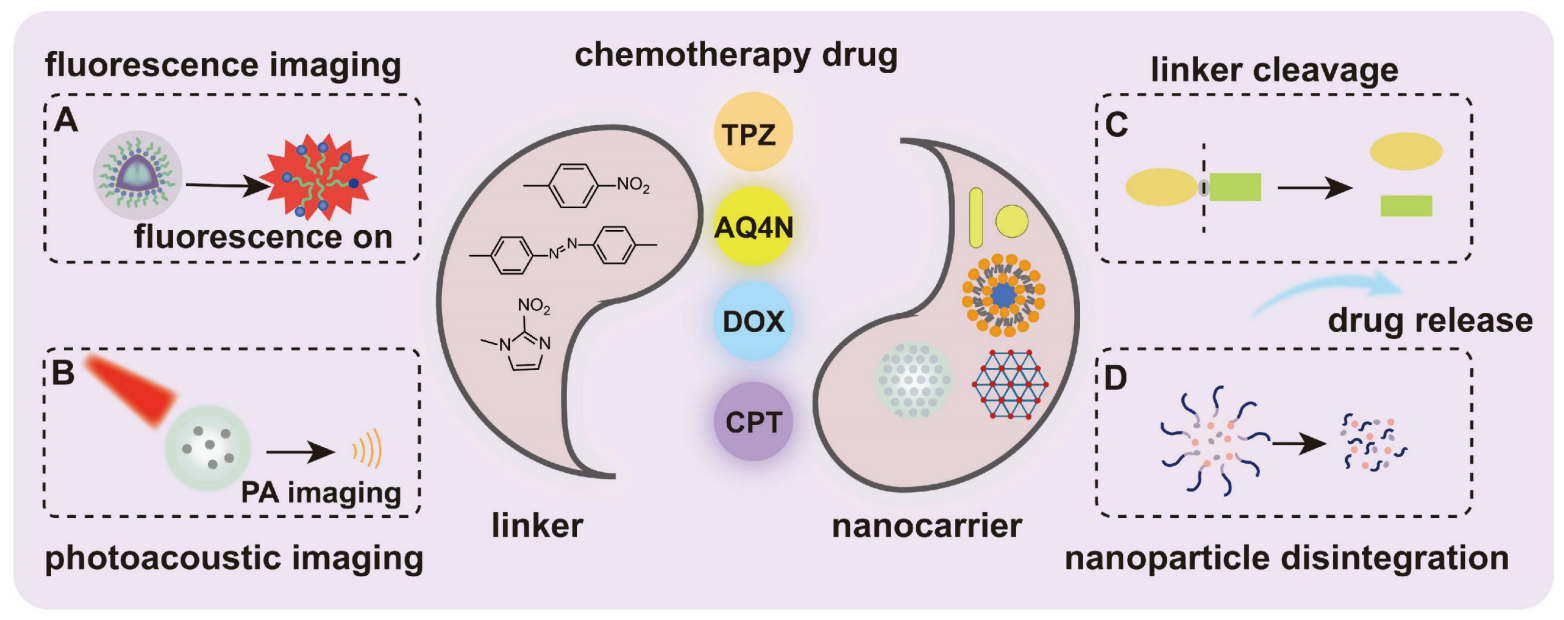
Schematic illustration of the hypoxia-responsive nanomedicine. (A) Fluorescence imaging. (B) Photoacoustic imaging. (C) Linker cleavage. (D) Nanoparticle disintegration. Once the nanocarrier is triggered by specific factors, such as hypoxia, it leads to the cleavage of the linker, the disintegration of the nanoparticle, and the subsequent release of fluorophores or drugs for disease diagnosis or treatment.

**Figure 2:**
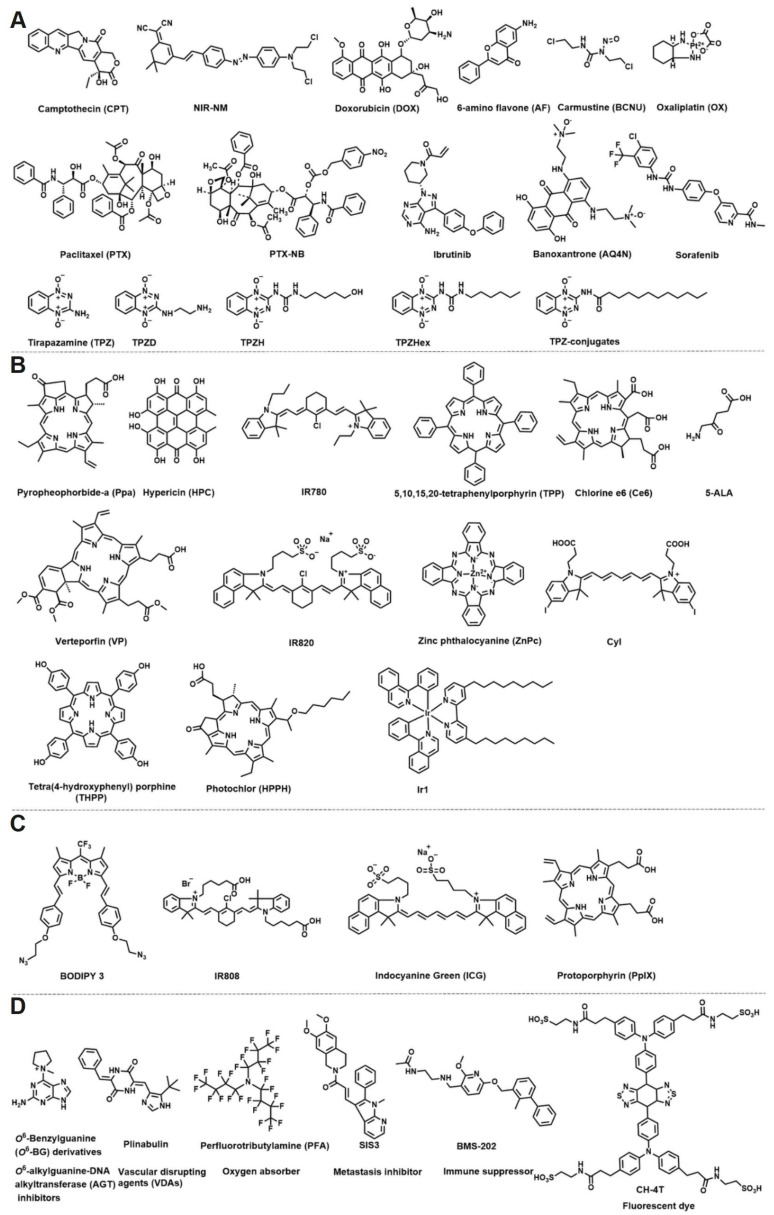
The small molecule structures in hypoxia-responsive nanoparticles. (A) Chemotherapy drug. (B) Photosensitizer. (C) Photothermic agent. (D) Other small molecular structures.

With the advancement of nanotechnology, the application of nanomaterials in cancer detection and treatment is becoming increasingly crucial. Hypoxia-activated chemical bonds, such as azobenzene 17, have been used to develop self-assembling nanoparticles that enhance drug permeability and accumulation at tumor sites. Additionally, functionalized nanomaterials serve as carriers for drugs or photosensitizers, facilitating targeted drug delivery 18, 19. The emergence of nanoparticles has enabled various imaging techniques, including fluorescence imaging and photoacoustic imaging, while simultaneously releasing drugs to combat tumors. This paper mainly discusses the research progress of nanoparticles utilizing different hypoxia-activated prodrugs in fluorescence imaging and cancer therapy (Figure 1). The structures of the drugs and other small molecules referenced in this paper are shown in Figure 2.

## Design strategies for hypoxia-responsive nanoparticles

Traditional chemotherapy and radiotherapy often struggle to precisely target tumor sites. In contrast, hypoxia-responsive nanoparticles can effectively target hypoxic regions within the tumor microenvironment 20, 21. Depending on their formation methods, nanoparticles can generally be classified into two main types: "self-assembly" and "physical encapsulation".

In the hypoxic tumor microenvironment, the expression of various reductases is upregulated 22, 23. Therefore, nanoparticles that activate and release imaging agents or drugs under hypoxic conditions can be designed to achieve the diagnosis and treatment of diseases. Self-assembled nanoparticles are usually formed through two primary methods 24-27: one is to connect hydrophilic fragments with hydrophobic fragments through hypoxia-responsive groups (such as azo groups, etc.) to form amphiphilic substances with hypoxic response. These amphiphilic substances are further polymerized to form nanoparticles with hydrophobic fragments as the core and hydrophilic fragments on the exterior. The second method requires modifying existing amphiphilic substances with hypoxia-responsive groups, so that non-responsive amphiphilic substances can react to hypoxia and subsequently self-assemble into nanoparticles in aqueous media.

**Figure 3:**
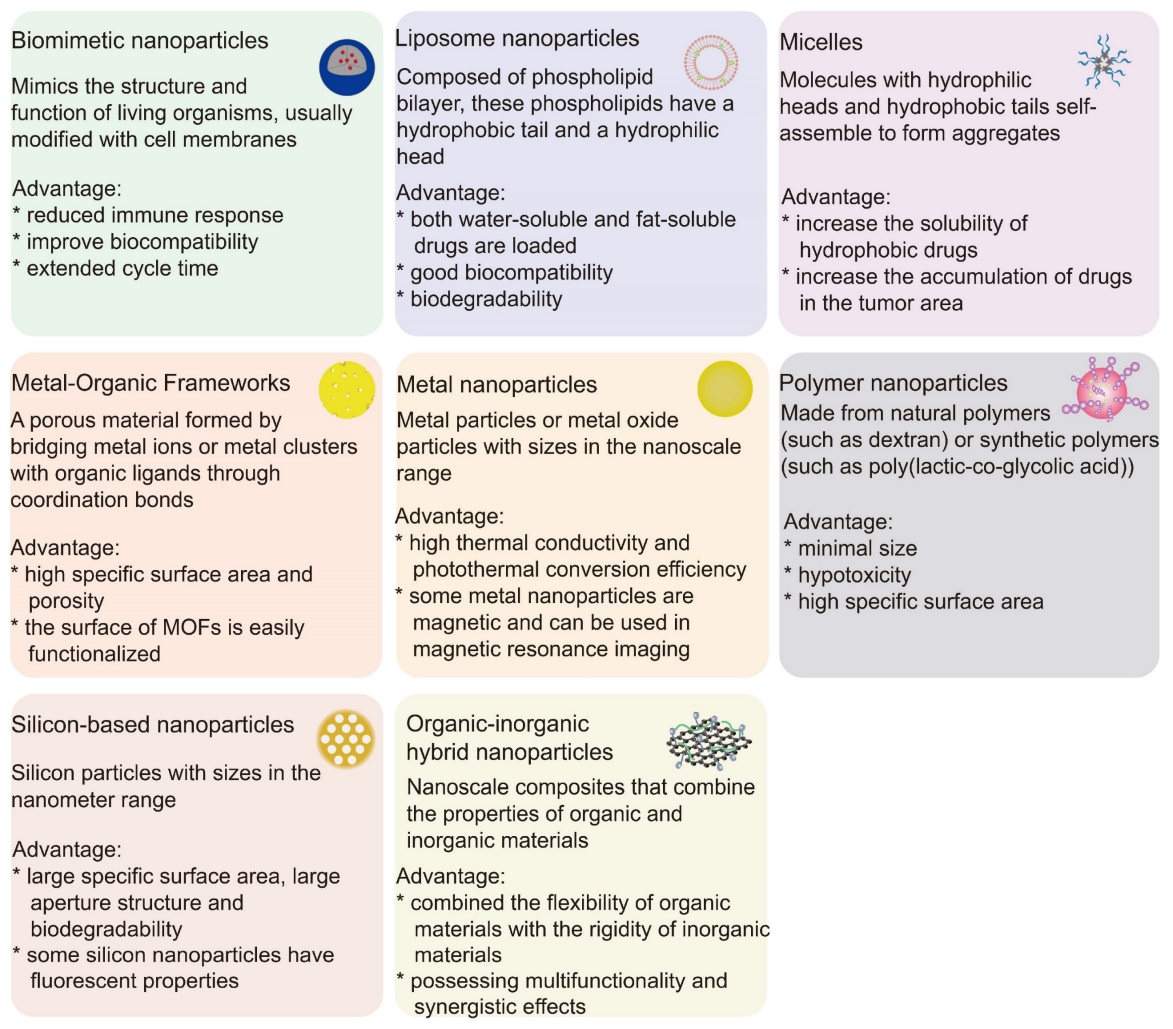
Different characteristics of nanoparticles and their advantages.

Since the oxygen concentration in tumors is much lower than that in normal tissues, the use of hypoxia-activated chemotherapy drugs is critical to minimize toxicity to normal tissue. However, treatment with a single drug may lead to resistance in tumor cells 28. Therefore, the concurrent delivery of hypoxia-activated prodrugs with imaging agents, photosensitizers, photothermal agents, glucose oxidase (GOx) or other chemotherapy drugs via physical encapsulation can not only kill tumor cells through multiple mechanisms of action, but also visualize drug release. The physical encapsulation strategy facilitates the design of nanoparticles with synergistic therapeutic effects, which can realize multimodal therapy and diagnosis, and is a good way to evaluate and improve the efficacy of drug therapy. Figure 3 lists common nanoparticles and their advantages.

## Hypoxia-responsive nanoparticles based on fluorescence imaging

**Figure 4:**
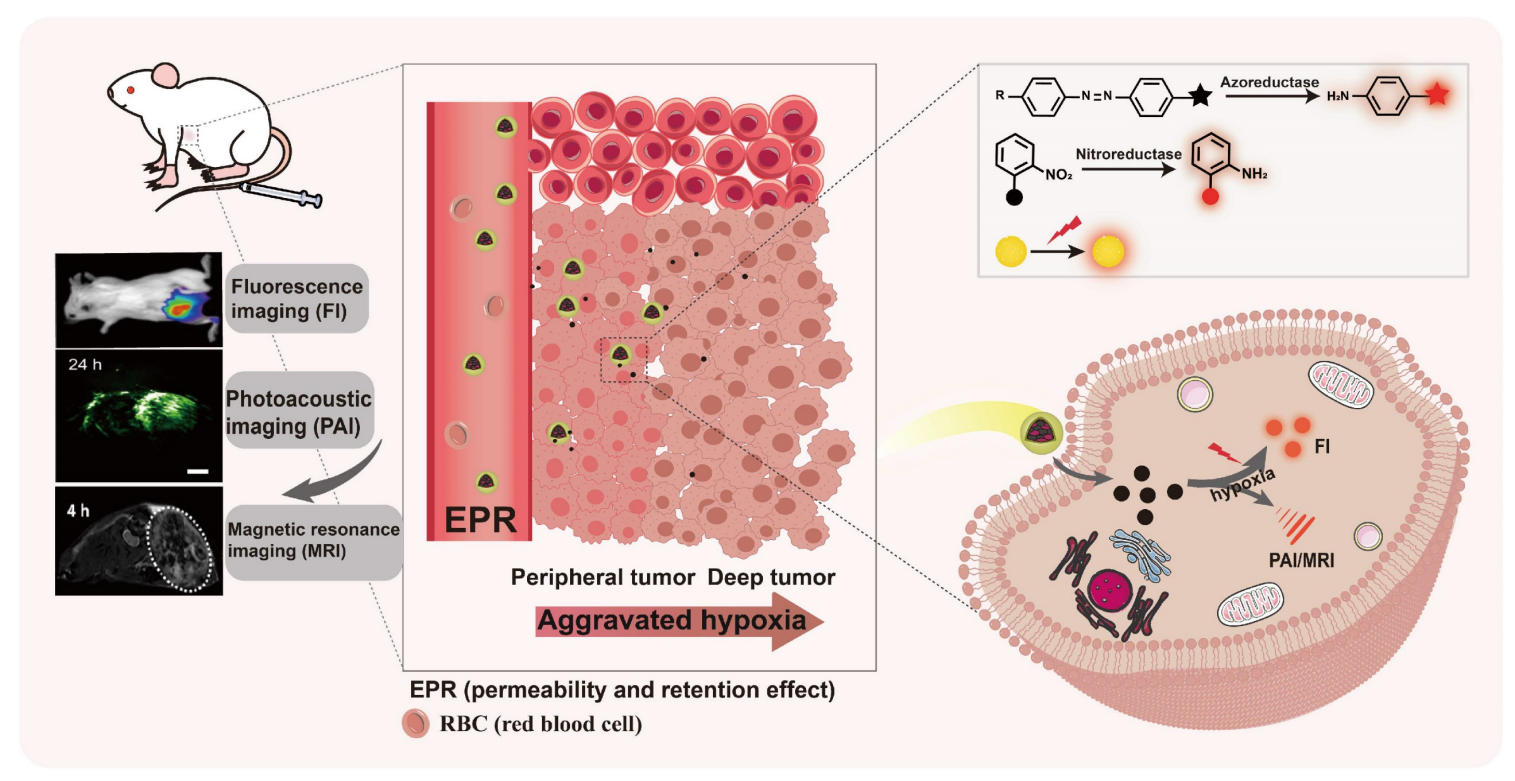
The scheme of hypoxia-responsive nanoparticles based on fluorescence imaging. Hypoxia-responsive nanoparticles utilize the EPR effect to deliver imaging agents to tumor sites, and provide a wealth of tumor-related information through fluorescence imaging and other fluorescence-based multimodal imaging technologies.

In recent years, the development of prodrugs has addressed the shortcomings of chemotherapy drugs with high toxicity and serious side effects on normal cells 29, 30. Real-time monitoring of drug release helps to accurately locate the lesion site, avoids the risks associated with improper drug dosing, and ultimately improves the therapeutic efficacy of the drug 31. The emergence of nanomaterials not only improves the biodistribution, solubility and half-life of some prodrugs 32, but also enables the delivery of a large number of imaging agents to tumor cells due to their large payload capacity, high signal strength and stability 33 (Figure 4). This section summarizes hypoxia-responsive nanoparticles based on fluorescence imaging (Table 1).

Small molecule prodrugs are obtained by linking the drug to fluorophores via linking groups 34. When encountering specific conditions in tumor cells, such as hypoxia, these small molecule prodrugs can release the corresponding drugs while simultaneously emitting fluorescence signals 35-37. The detection of drug release can be achieved through changes in fluorescence signals, and this design also prevents the drug from harming normal tissues. Nitrogen mustard (NM) is a potent antineoplastic agent; however, its administration *in vivo* inevitably leads to significant toxicity in healthy cells. Li *et al*. 38 described a nanoscale drug delivery system based on hybrid liposomes, designated FA-lip@NIR-NM. Nitrogen mustard was first conjugated with a near-infrared fluorophore (a dicyanoisophorone derivative) through an azo bond to form the prodrug NIR-NM. This prodrug was then encapsulated within liposomes composed of DSPE-PEG2K-FA, soy lecithin, and cholesterol, resulting in the nanoparticle prodrug FA-lip@NIR-NM. In the presence of azo reductase, the azo bonds of the prodrug were cleaved, producing compounds with strong electron-donating groups that activated fluorescence and released drugs. Significant fluorescence signals were detected under hypoxic conditions (2% O_2_) compared to normoxic conditions (21% O_2_). Furthermore, mice treated with FA-lip@NIR-NM showed stronger fluorescence signals at the tumor site than those injected with NIR-NM, and the significant signal persisted for 48 hours. FA-lip@NIR-NM offers significant benefits, including enhanced tumor targeting and prolonged *in vivo* circulation, which facilitate the tracking of active chemotherapeutic agent release and enhance the therapeutic index.

Employing a similar strategy, Yuan* et al*. 39 developed an amphiphilic block copolymer, PCL_3k_-TPE-Azo-PEG_5k_, using azo bonds that respond to azo reductase. The azobenzene group was directly conjugated to tetraphenylethylene (TPE) to form TPE-azo, which connected hydrophilic (PEG) and hydrophobic (PCL) chain segments to obtain amphiphilic block copolymer. This synthesized compound could self-assemble in PB solution to form rod-like micelles and encapsulated DOX. The presence of the azo group inhibited the AIE aggregation of TPE, rendering the micelles non-fluorescent. However, upon the introduction of a reducing agent (azo reductase or Na_2_S_2_O_4_), the azo bond was cleaved, leading to micelle collapse and activation of the AIE effect. This sequence of events restored the fluorescence of TPE, with the fluorescence intensity increasing in conjunction with the release of the drug.

Wang *et al.* 40 designed a drug-loaded micelle, mPEG_2k_-ADP-Azo@DOX, utilizing DOX as a chemotherapy drug and Aza-BODIPY (ADP) as a fluorophore. They directly linked the near-infrared fluorescent molecule Aza-BODIPY with azobenzene to form the fluorescent probe ADP-Azo, followed by the introduction of two reactive alkyne groups. The mPEG_2k_-ADP-Azo was synthesized through a copper-catalyzed azide-alkyne cycloaddition (CuAAC) reaction, and subsequently encapsulated DOX to form drug-carrying micelles. The azo bond initially kept the fluorescence of Aza-BODIPY in the “OFF” state; however, in the presence of azo reductase, the azo bond was cleaved and near-infrared fluorescence was activated. The fluorescence intensity gradually increased within the range of 700-750 nm. Based on this, drug-loaded micelles could control and monitor drug release in real-time. Additionally, Wang *et al.* 41 developed a live cell fluorescence imaging agent, Zr-MOF@PPa/AF@PEG, utilizing 6-aminoflavone (AF) as a chemotherapeutic agent and Pyropheophorbide-a (Ppa) as a photosensitizer to realize the anti-tumor effects of cascade photodynamic therapy (PDT)-chemotherapy (CT). The loading of Ppa onto nanomaterials addressed the issue of self-polymerization. Zr-MOF@PPa/AF@PEG exhibited strong fluorescence emission at 550 nm under the slightly acidic tumor microenvironment. Cellular imaging results indicated that, after a 15-minute treatment, the red fluorescence in cells treated with nanoparticles was stronger than that in cells treated with free Ppa.

Furthermore, utilizing the CuAAC “click” reaction, Wang *et al*. 42 synthesized a novel amphiphilic block copolymer, PPEGMA_14_-ADP-Azo-PBzMAx, in 2022. Upon reaction with azoreductase, the nanoparticles underwent elimination of the aggregation-caused quenching (ACQ) effect and subsequent dissociation, resulting in drug release alongside a significant enhancement of near-infrared (NIR) fluorescence at 710 nm. Additionally, the nanoparticles exhibited increased fluorescence intensity under hypoxic conditions (1% O_2_) compared to normal oxygen conditions (16% O_2_). This was the first NIR fluorescent nanoprobe synthesized by RAFT-mediated PISA strategy, enabling simultaneous cell imaging and drug release.

In addition to utilizing the reduction of azo groups under hypoxic conditions, scientists often select nitro groups as reducing groups under hypoxic conditions. Zhang *et al*. 43 fabricated Cy7-NO_2_/HPC/TPZ NPs by integrating the photosensitizer hypericin (HPC), the hypoxia-activated prodrug tirapazamine (TPZ), and the hypoxia detection probe Cy7-NO_2_. These nanoparticles demonstrated high penetrability, and due to different oxygen requirements, HPC and TPZ achieved a synergistic therapeutic effect by combining chemotherapy with PDT. Furthermore, the NPs retained the necrotic targeting ability of HPC, which facilitated profound tumor infiltration and enhanced the accumulation of NPs within the tumor microenvironment. Fluorescence imaging of tumors of varying sizes confirmed that the hypoxic status in different tumors could be visualized by monitoring nitroreductase levels. Experiments conducted both *in vivo* and *in vitro* showed that image-guided flexible adjustments could optimize tissue penetration and improve treatment outcomes under different treatment regimens.

Scientists also used hypoxic detection probes to image myocardial hypoxia, thereby expanding their range of applications. Fan *et al*. 44 developed a liposome-based nanostructure, Pep/BDP-NO_2_@Lip, in which the liposome was functionalized with a peptide (GGGGDRVYIHPF) and encapsulated nitrobenzene-substituted BODIPY. The styryl substitution resulted in a redshift of the maximum emission peak. The presence of 2-nitrobenzene induced electron transfer, leading to fluorescence quenching. In the presence of nitroreductase (NTR), the nitro group underwent reduction to an amino group and the electron transfer process was suppressed. Consequently, fluorescence intensity was significantly enhanced, and a distinct emission peak was observed at 713 nm. The authors further utilized the nanoprobe for real-time hypoxia imaging in myocardial ischemic mouse models, confirming that the nanoprobe successfully entered hypoxic heart cells and realized the detection of intracellular NTR.

**Figure 5:**
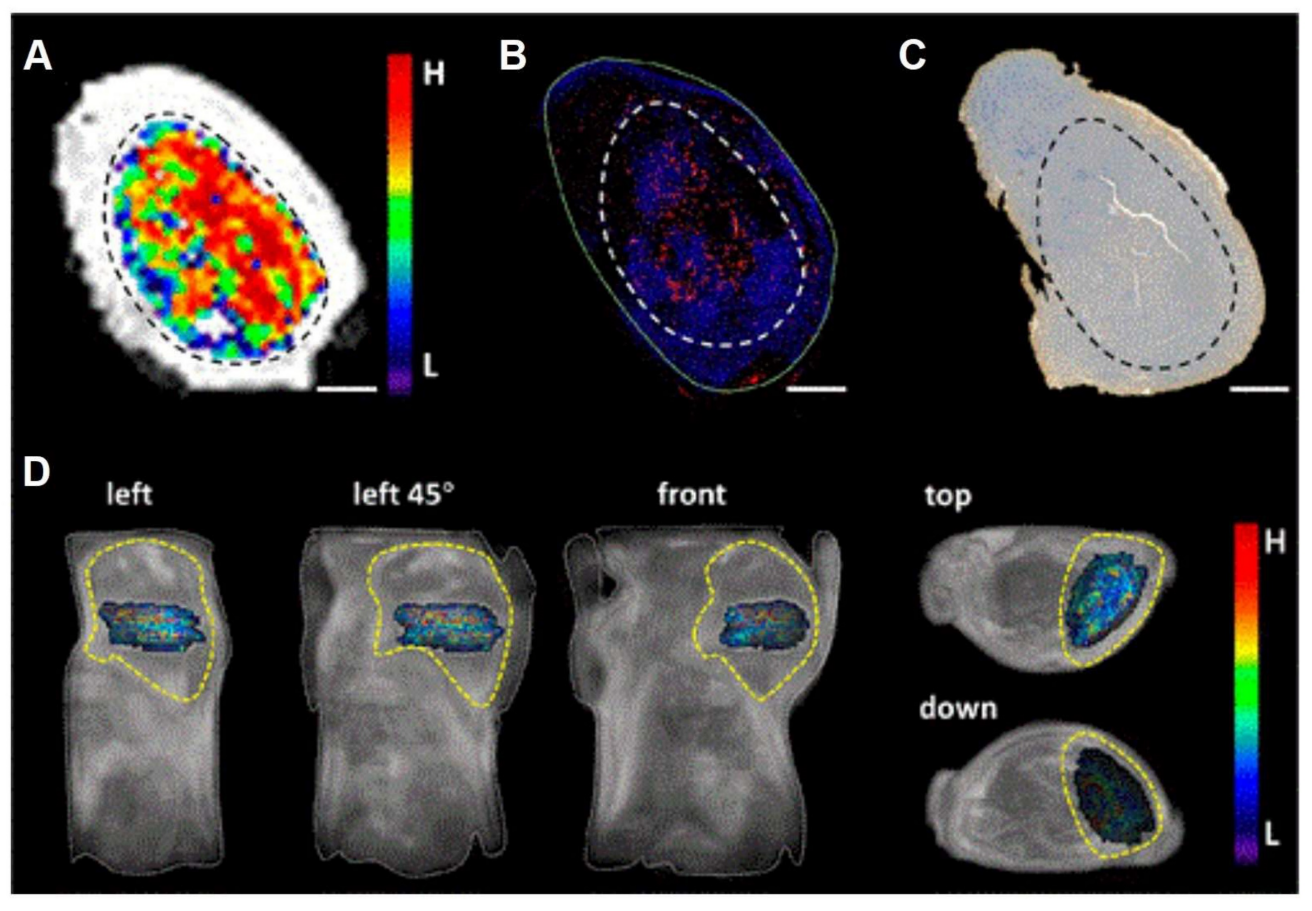
(A) UIO-Pimo showed hypoxic areas within the tumor by MRI difference value method. Immunofluorescence of HIF-1α antibody (B) and HE staining of Pimonidazole (C). (D) The MRI difference value method was used to show the distribution of hypoxia *in vivo*. Adapted with permission from 46, copyright 2021, American Chemical Society.

HOISNDs nanoparticles developed by Fu *et al.* 45 could effectively enhance fluorescence and magnetic resonance imaging of tumor sites, facilitating accurate diagnosis and treatment of cancer. Zhou *et al*. 46 also created a dual-modality imaging probe, UIO-Pimo, which consists of hypoxia-triggered self-assembled ultra-small iron oxide (UIO) nanoparticles and responsive fluorescent dyes (NBD). Both *in vivo* and *in vitro* experiments demonstrated that UIO-Pimo enhanced the fluorescence of NBD by incorporating self-assembly through a hydrophobic environment under hypoxic conditions. At the same time, *in vivo* imaging validated the ability of the probe to generate stronger MRI signals in the tumor area. UIO-Pimo not only fully assembled, but also realized dual-mode imaging and established an MRI differential analysis method to visualize the three-dimensional distribution of tumor hypoxia. Additionally, UIO-Pimo exhibited significant penetration and accumulation efficiency in hypoxic regions, suggesting its potential as a targeted drug delivery platform for hypoxic conditions (Figure 5).

**Figure 6:**
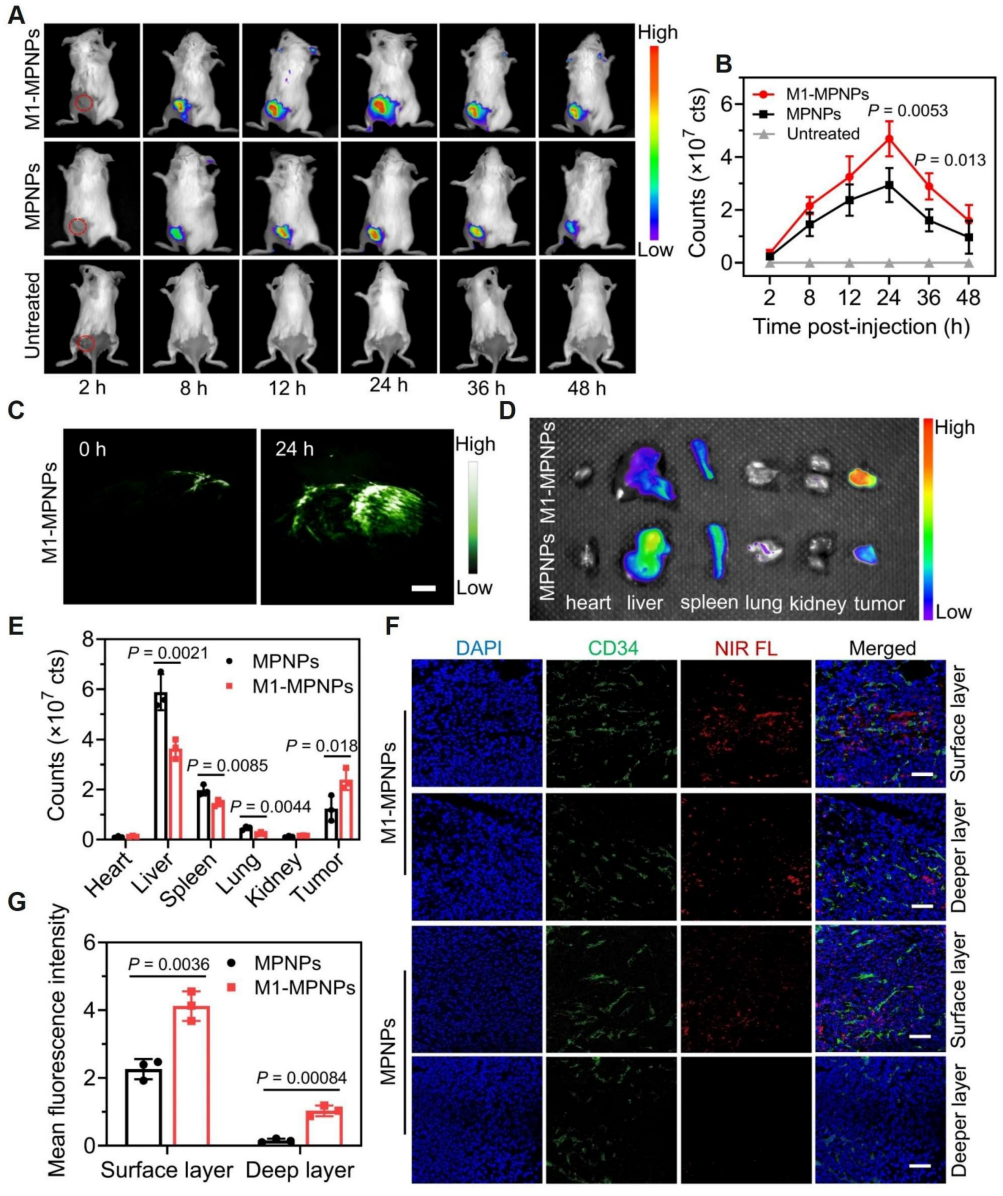
Dual-mode imaging of M1-MPNPs. Fluorescence images treated with MPNPs or M1-MPNP were obtained at mouse tumor sites (A), major organs and tumors (D), and mouse tumor sections (F). (C) PA images of tumor site after treatment with M1-MPNPs. The corresponding fluorescence intensity graphs of (A), (D), (F) are shown in (B), (E), (G). Adapted with permission from 47, copyright 2023, Springer Nature.

To achieve complementarity between fluorescence imaging and photoacoustic (PA) imaging, Kang *et al*. 47 developed a self-accelerated nanoplatform M1-MPNPs by combining the chemotherapy drug paclitaxel (PTX) with aggregation-induced emission luminogens (AIEgens). The AIEgens used were TPE-TT and MTPE-TT. Compared with TPE-TT (609 nm), the fluorescence brightness and PA intensity of the methoxy-substituted MTPE-TT (624 nm) were further increased, and the PDT performance was also increased due to the enhanced ICT effect. The nanoparticles were camouflaged with macrophage membranes to create a tumor-targeting therapeutic drug, M1-MPNPs. The fluorescence intensity of M1-MPNPs peaked approximately 24 hours post-injection in 4T1 tumor-bearing mice. Notably, even after 48 hours, the near-infrared fluorescence signal remained discernible at the tumor site. *In vivo* PA imaging of M1-MPNPs revealed that the amplitude of PA signal reached its maximum 24 hours after intravenous administration. Through reasonable design, the authors successfully leveraged the complementary advantages of fluorescence and PA dual-mode imaging to sensitively map tumor sites and provide more accurate diagnostic information (Figure 6), providing references for the development of tumor immunotherapy drugs.

Similarly, Qu *et al*. 48 also developed a dual-modality nanoparticle for fluorescence and photoacoustic imaging. They prepared PCM@Lip/IT NPs using a eutectic mixture of lauric and stearic acids as the core, encapsulating the chemotherapeutic drug TPZ and the photosensitizer IR780. This eutectic mixture, formed from natural fatty acids, exhibited a sharp melting temperature. When irradiated with near-infrared laser light, IR780 induced heating of the nanoparticle's lipid membrane, leading to the release of TPZ and IR780. The photoacoustic signal intensity was found to be linearly correlated with the concentration of IR780. Moreover, the PA signal and fluorescence intensity increased over time following the intravenous injection of PCM@Lip/IT NPs. The mitochondrial targeted phototherapy nanosystem prepared by the authors using IR780 in combined with TPZ is expected to alleviate the adverse effects associated with conventional chemotherapy and enhance the overall anti-tumor effect.

In addition to the aforementioned methods, scientists also combine fluorescence imaging with X-ray computed tomography (CT) and positron emission tomography (PET), using X-rays or PET tracers containing radionuclides to address the limited penetration depth of fluorescence imaging. However, most nanoparticles currently integrating CT or PET with fluorescence imaging are non-hypoxic-responsive. For example, Zhang *et al.* 49 synthesized a nanoprobe for FL/PET dual-modality imaging using the fluorescent dye CH-4T and the PET radionuclide ^64^Cu, named CH-4T/SLB-MSN-MDOT/^64^Cu^2+^. This nanoprobe significantly enhanced the fluorescence of CH-4T. In the A431 tumor model, PET imaging showed clear contrast. Zeng *et al.* 50 combined gold nanospheres with mesoporous silica nanocarriers (MSNs) and encapsulated ICG to obtain nanoparticles, Au@MSNs-ICG, which exhibited fluorescence and CT dual-modality imaging signals. At present, there is a scarcity of research on hypoxia-responsive nanoparticles that leverage these two types of dual-modality imaging, necessitating further investigation in the future.

## Hypoxia-responsive nanoparticles for therapy

**Figure 7:**
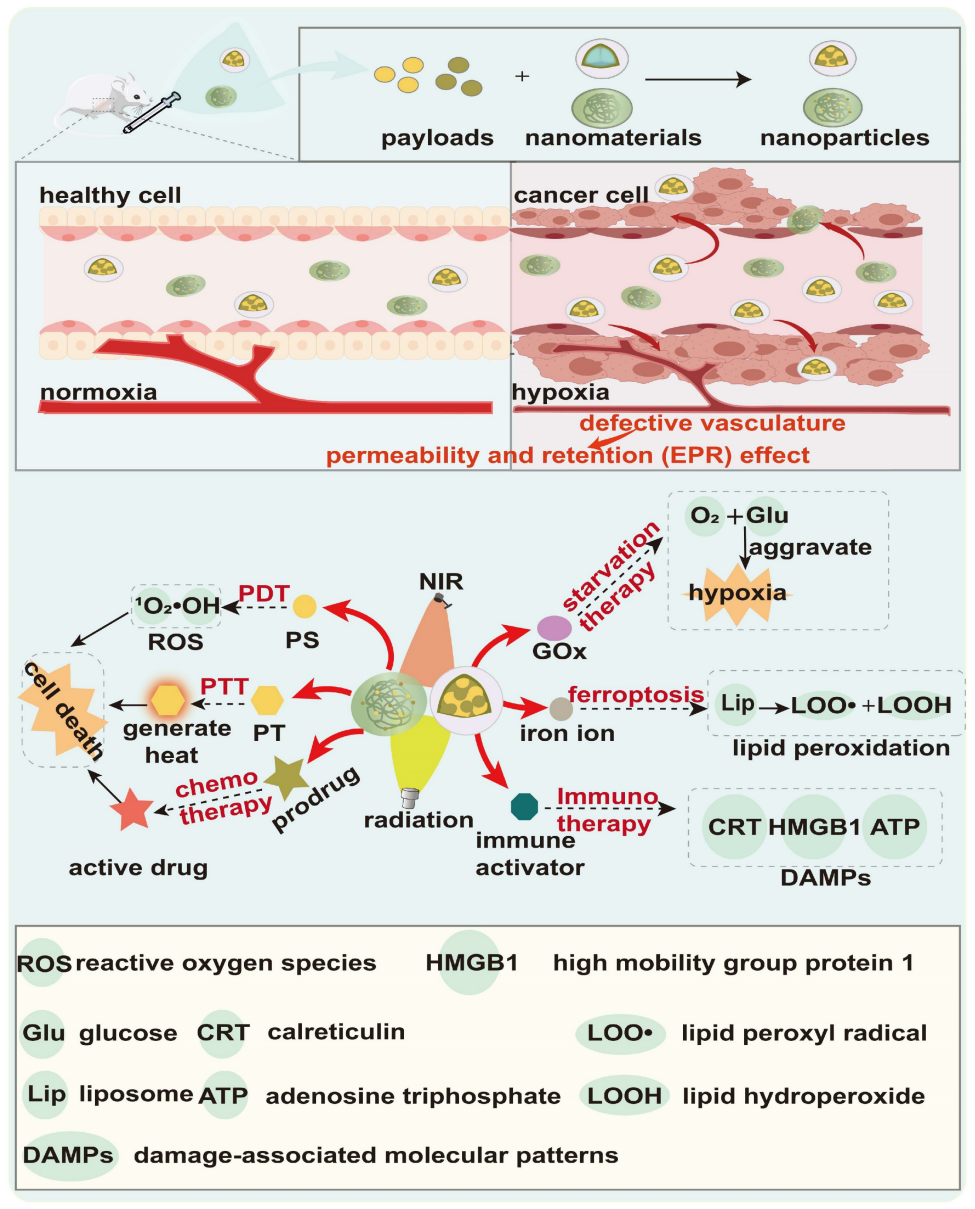
The scheme of hypoxia-responsive nanoparticles for therapy. Hypoxia-responsive nanoparticles can encapsulate a variety of therapeutic drugs and synergistically exert anti-tumor effects through various methods, including photodynamic therapy (PDT), photothermal therapy (PTT), chemotherapy, ferroptosis, immunotherapy, and starvation therapy.

With advancements in treatment, mortality rates for some cancers, such as kidney cancer, have gradually decreased in recent years. However, cancer remains a leading cause of death worldwide 51. According to global cancer statistics, the number of cancer patients continues to increase 1, 2, which further emphasizes the need for improved treatment strategies. Chemotherapy, a common clinical treatment for cancer, relies on the delivery of chemical drugs to cancer cells. Chemotherapeutic drugs can exert a therapeutic role only when they reach a sufficient concentration; however, they can also cause serious adverse effects, including neuropathy, cardiomyopathy, and congestive heart failure 52. Generally, the stronger the efficacy of anticancer drugs, the more toxic they are 53, which further limits their clinical application. Nanotechnology offers a promising solution that allows chemotherapeutic drugs to be attached to nanomaterials through various methods, such as physical capture, non-covalent adsorption, or covalent bonding with degradable or non-degradable bonds 54. These modifications enhance the drug's solubility, stability, and biocompatibility, ultimately improving the effectiveness of cancer treatment. Given the limited sensitivity of pH and enzyme-responsive strategies in the early stages of tumors 55, and the presence of hypoxia at different stages of tumors, this paper reviews hypoxia-responsive nanoparticles for therapy (Figure 7).

### Nitro-based nanoparticles

In the specific pathological environment of hypoxia, there is a significant upregulation of reductase enzymes, including nitroreductase, azoreductas, and quinone reductase 56-58. Hypoxia-sensitive bioreductive molecules, such as nitroaromatics, azo derivatives and quinones have been widely used in hypoxia-responsive nanosystems. Under hypoxic conditions, nitro derivatives undergo a single-electron reduction process mediated by enzymes, which converts the nitro group into amine or hydroxylamine derivatives 59. Nitroaromatic compounds mostly exhibit minimal or no inherent pharmacological activity and are often used in combination with various chemotherapeutic agents 60. Their applications in self-assembling nanocarriers are particularly noteworthy; in this context, the nitro group acts as a hypoxia-sensitive trigger that, upon reduction, triggers the disintegration of the nanoparticles and facilitates the release of encapsulated drugs, thereby enabling the treatment of cancer. This section provides an overview of reduction-mediated drug nanoparticles based on the nitro group (Table 2).

Because traditional drug delivery systems face the challenge of achieving optimal drug concentrations at tumor sites, scientists have explored various stimuli-responsive systems. Sun *et al*. 61 developed a nanoparticle featuring a 4-nitrobenzyl group as a nitroreductase (NTR)-responsive component. First, hydrophobic alkylated tetraphenylene (TPE), hydrophilic polyethylene glycol (PEG) chains, and 4-nitrobenzyl were connected by 2,6-bis(hydroxymethyl)-*p*-cresol, resulting in the formation of micelle TNP in aqueous solution. This was followed by the encapsulation of the hydrophobic drug doxorubicin (DOX) within its hydrophobic chamber. In the presence of NADH, the 4-nitrobenzyl group of TNP was reduced by NTR, leading to the degradation of TNP micelles and subsequent release of DOX. TNP micelles demonstrated remarkable sensitivity and specificity in response to NTR, with a detectable concentration as low as 2.6 ng·mL^-1^. The MTT assay revealed that free DOX was highly toxic to both cancerous and normal cells, while TNP@DOX micelles exhibited significant toxicity primarily towards cancer cells. This selectivity can be attributed to the low levels of NTR present in normal cells. The degradation of the micelles was consistent with the release of TPE derivatives. Leveraging the aggregation-induced emission (AIE) phenomenon of TNP, the researchers successfully monitored the degradation of TNP aggregates, thereby enabling the controlled and monitored release of intracellular DOX.

Also choosing *p*-nitrobenzyl as the hypoxia-responsive component, Zhang *et al*. 62 coupled AP-NC with the mPEG-PPLG copolymer to synthesize mPEG-PLG-NC. This construct self-assembled in aqueous solution and encapsulated the anticancer drug DOX, resulting in drug-loaded nanoparticles designated as PPGN@DOX. Under hypoxic conditions, the *p*-nitrobenzyl groups were reduced to *p*-aminobenzyl groups, which triggered the release of DOX. PPGN@DOX exhibited significant anticancer activity both *in vivo* and *in vitro*. Furthermore, animal experimental data indicated that PPGN@DOX enhanced the bio-distribution of DOX, amplified its anticancer effects, and reduced the toxic side effects on healthy cells and tissues.

Hypoxia-responsive drugs can be delivered not only via micelles; scientists have also explored liposome-based drug delivery systems. Li *et al*. 63 synthesized liposomes that do not respond to stimulation (LPs) alongside those specifically responsive to hypoxic conditions (HR-LPs). HR-LPs were created by integrating a nitroimidazole derivative into the phospholipid bilayer of the liposomes. Subsequently, DOX-LPs and DOX-HR-LPs were obtained by encapsulating DOX within the liposomes. Following 12 hours of hypoxic treatment, the cumulative release of DOX from the DOX-LPs was minimal, indicating that LPs were stable under physiological conditions. In contrast, the hypoxia-induced reduction of nitroimidazole derivatives resulted in the disruption of DOX-HR-LPs, which significantly increased the release of the drug. The DOX-HR-LPs exhibited enhanced antitumor efficacy and reduced systemic toxicity, making them promising candidates for the treatment of hypoxic tumors.

The hypoxic heterogeneity of tumors poses a challenge to the clinical application of hypoxia-activated prodrugs. To overcome this problem, Zhou *et al*. 64 developed an innovative block copolymer polyprodrug, PEG_113_-*b*-P(CPTNMA_6_-*co*-TPPMA_1_), by integrating PDT with a hypoxia-activated prodrug. This copolymer self-assembled into spherical micelles (PPMs) using the nanoprecipitation method. The compound 5,10,15,20-tetraphenylporphyrin (TPP) exerted PDT effects in oxygen-rich regions under light, converting O_2_ into ^1^O_2_ and inducing apoptosis, while simultaneously transforming oxygen-rich regions into anoxic regions. In hypoxic areas, the nitroimidazole linker was reduced by the overexpression of nitroreductase, leading to the release of free camptothecin (CPT). By combining the effects of PDT and chemotherapy, PPMs exhibited a powerful synergistic antitumor effect. This combination not only addresses the limitations of single PDT treatment or hypoxia-activated prodrugs, but also effectively mitigates the side effects commonly associated with traditional chemotherapeutic agents.

### Azobenzene-based nanoparticles

Similar to nitro groups, azo groups are also nitrogen-containing active groups that have received increasing attention in hypoxic response systems 65. In particular, azobenzene compounds, due to their hypoxia-activated cleavage properties, can act as substrates for azo reductase to initiate molecular cleavage of derivatives. In the hypoxic tumor microenvironment, azo compounds are reduced to amino derivatives by azoreductase, which is one of the important strategies for designing hypoxia-responsive nanotherapeutics 36, 66. Unlike the mechanism by which nitro responds to hypoxia, azobenzene is decomposed into aniline by accepting four electrons 67, which makes azobenzene more effective as a hypoxia-sensitive site. This section summarizes the azobenzene-based nanoparticles (Table 3).

Li *et al.* 68 developed a novel nanoparticle, HACB/BCNU NPs, which used the azobenzene group as a hypoxia-responsive linker. Building on the innovative use of azobenzene in drug delivery, Yang *et al*. 69 further explored this strategy by developing a size-adjustable nanosystem, HCHOA. They coupled the photosensitizer chlorine e6 (Ce6) to human serum albumin (HSA) to form HC, and likewise conjugated an oxaliplatin prodrug with HSA to create HO. These components were then covalently connected via an azobenzene bridge to construct HCHOA. Under normal oxygen partial pressure, HCHOA exhibited a diameter ranging from 100 to 150 nm, resulting in prolonged blood circulation and enhanced tumor accumulation. Upon exposure to hypoxia, the azobenzene groups within HCHOA were cleaved by reductase, resulting in the rapid disintegration of the nanosystem into ultrasmall HC and HO complexes with diameters less than 10 nm. The decomposed small nanoparticles significantly improved penetration ability within tumors compared to HCHOH nanoparticles that lacking an anoxic response. At the same time, due to the dissociation of HCHOA, the fluorescence of Ce6 was restored, making it suitable for biological imaging. Moreover, the photoactivity of Ce6 was activated, increasing the concentration of singlet oxygen and facilitating the synergistic effects of PDT and chemotherapy. The authors achieved the goal of adjustable nano size by reducing azo at the hypoxic site, thus improving the therapeutic effect on tumor.

Ma *et al.* 70 utilized p-aminoazobenzene as a linker and the prodrug IR808-S-S-PTX as a hydrophobic block, which was conjugated to glycosylated PEG, resulting in the formation of the amphiphilic polyprodrug conjugate Glu-PEG-Azo-IR808-S-S-PTX. Under hypoxic conditions, the azo bond underwent cleavage, releasing the IR808-S-S-PTX moiety, which was then converted to PTX and IR808 by glutathione (GSH), thereby exerting therapeutic effects on tumors. Fluorescence imaging studies demonstrated that these micelles could selectively aggregate in tumor lesions and the mitochondrial compartment, effectively inhibiting tumor growth and metastasis without causing serious systemic toxicity. The targeting of glucose transporter 1 (GLUT1) and the hypoxia-responsive characteristics of the micelles substantially enhanced the therapeutic efficacy of drugs.

**Figure 8:**
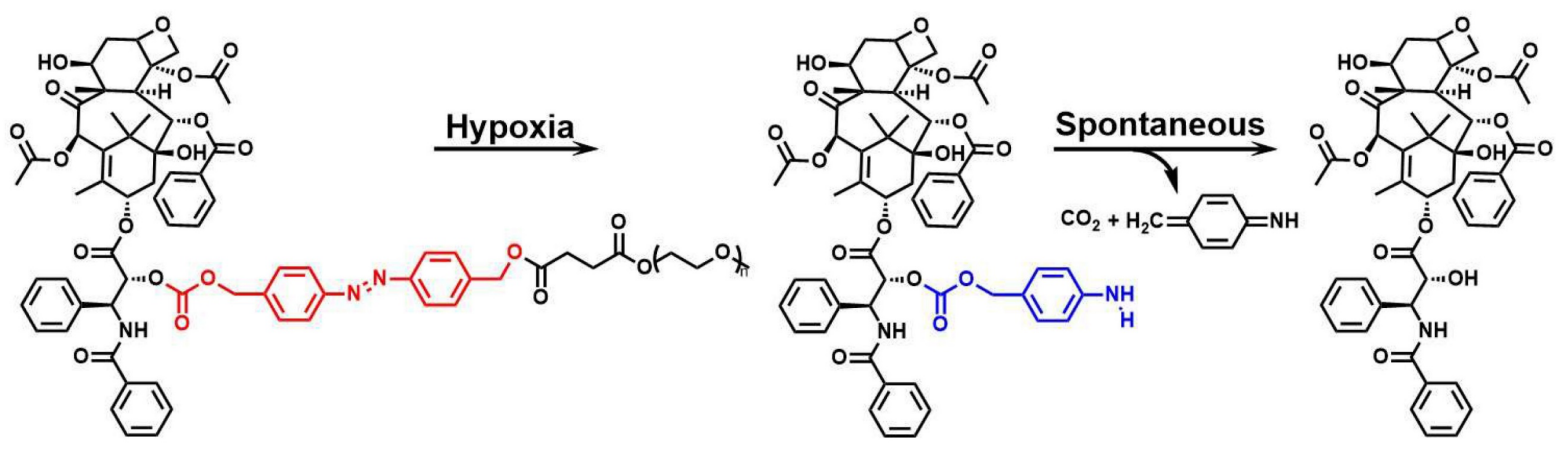
Reaction mechanisms of azobenzene compounds. Adapted with permission from 71, copyright 2022, American Chemical Society.

Employing a similar strategy, Hao *et al*. 71 synthesized the prodrug PAPs by introducing an azo bond as a bridging linker that connected paclitaxel (PTX) with methoxy-PEG (mPEG), which then self-assembled into nanoparticles known as PAP NPs. Under hypoxic conditions, the azo group underwent lysis, triggering the release of PTX and eliciting cytotoxicity in cancer cells (Figure 8). PAP NPs with shorter mPEG chains have better cell safety and produce cytotoxicity when PTX is released in a hypoxic environment, thereby mitigating off-target toxicity.

Although both utilize the hypoxia-triggered azo bond release mechanism for targeted drug delivery, Hao *et al.* 71 focused on self-assembled nanoparticles for direct drug release, while Yan* et al.* 72 developed a gated smart MSN system for controlled drug release in response to hypoxia. The azobenzene polymer was deposited on the surface of the MSN, and the amphiphilic copolymer Pluronic F68 was subsequently added to form MSN/pDAB/F68. Due to the reducibility of azobenzene, these nanoparticles exhibited high sensitivity to low oxygen concentrations. Selecting coumarin 6 and Rhodamine B as the donor and acceptor of fluorescence resonance energy transfer (FRET), respectively, the researchers demonstrated the hypoxic response release of model fluorophores within MCF-7 cells. In addition, the authors loaded the photosensitizer Ce6 into gated microsphere and verified its cytotoxicity under hypoxic conditions. Gated MSNs enable the treatment of tumor cells while avoiding the premature release of drugs, thereby broadening the potential applications of microspheres in pharmaceutical and biomedical fields. Furthermore, Chen *et al.* 73 synthesized DOX@Biotin-SAC4A by leveraging the active targeting capability of biotin, thus further innovating the design of hypoxia-responsive nanoparticles.

### Nanoparticles based on benzotriazine nitrogen oxides

It has been found that when tumors exceed their vascular supply, they exhibit tumor heterogeneity 74 and the formation of hypoxic cells with diverse oxygen levels. Tirapazamine (TPZ), a hypoxia-activated prodrug, generates transient oxidizing free radicals through reductase metabolism, which leads to the destruction of DNA strands under hypoxic conditions 75-77, thereby increasing toxicity to cancer cells. The combination of TPZ with other therapeutic agents can enhance the efficacy of TPZ, although it may also result in side effects, such as muscle spasms 78. Additionally, the limited penetration and rapid clearance of TPZ at the tumor site hinder its therapeutic effectiveness. To solve this problem, researchers have improved the efficacy of TPZ by combining it with nanomaterials. This section summarizes the therapeutic methods and effects of nanoparticles based on benzotriazine (Table 4).

Ajnai *et al*. 79 utilized gold nanoparticles (GNPs) in conjunction with TPZ to augment drug accumulation within tumors through the enhanced permeability and retention (EPR) effect, employing bovine serum albumin (BSA) as a binding agent. In MKN45 xenograft models, GNPs-TPZ demonstrated improved tumor cytotoxicity and superior tumor-targeting capabilities compared to TPZ alone. In addition, no significant changes were observed in the blood biochemical parameters of mice treated with GNPs-TPZ. Taken together, these results indicate that GNPs-TPZ enhances tumor targeting and exhibits a more effective therapeutic effect.

Like TPZ, its derivatives also have good potential for hypoxic tumor therapy. However, TPZD is characterized by poor biocompatibility and low availability. Zhao *et al*. 80 synthesized the zwitterionic polymer BCP-TPZ by chemically coupling the block copolymer BCP with TPZD. BCP was initially produced by reversible addition-fragmentation chain transfer (RAFT) polymerization. The FPMA fragment contained an active aldehyde group, which was covalently linked to the TPZ derivative (TPZD) via an imine bond. Imaging of BCP-TPZ labeled with FITC revealed that these micelles could efficiently deliver TPZ derivatives and retain them in the cytoplasm. The cleavage of imine bonds under acidic conditions facilitated the sustained release of TPZD in the tumor region, thus enhancing the therapeutic effect. Xu *et al*. 81 developed the derivative TPZH and conjugated it with poly(L-glutamic acid) (PLG) to form nanoparticles known as TPZH-NPs. The experiment showed that the presence of esterase enabled the sustained release of TPZH from the nanoparticles and enhanced its accumulation in tumors. Lv *et al.* 82 also conducted similar studies.

Tumor heterogeneity often affects the effectiveness of hypoxia-activated prodrugs. To address this, scientists have explored the treatment of hypoxic tumors using a combination of tumor deoxygenation and hypoxia-activated prodrugs. Chen *et al*. 83 developed a nanomedicine that employed an UiO-66 metal-organic framework (MOF) as a drug carrier, which encapsulated TPZ and perfluorotributylamine (PFA) in a polydopamine (PDA)-coated porous nanomaterial, with PFA serving as the oxygen absorber. Upon entering tumor tissue, the PFA within the nanomedicine consumed oxygen, exacerbating tumor hypoxia and triggering the activation of TPZ, thereby inducing tumor cell apoptosis. The combination of TPZ, PFA and nanoparticles enhanced permeability and retention. Therefore, following administration via the caudal vein, TPZ/PFA@UiO-66@PDA demonstrated a tendency for tumor-specific accumulation, subsequently exerting a suppressive effect on tumor proliferation.

Unlike the oxygen scavenging property of PFA, thrombin exacerbates hypoxia by initiating vascular infarction. Ma *et al*. 84 selected a metal-organic framework (MOF) and functionalized its surface with folic acid (FA) to enhance tumor targeting. They added thrombin and TPZ into the MOF, producing nanoparticles called Th-TPZ@MOF-FA. These nanocarriers exhibited pH-responsive release behavior and were able to selectively release drugs into tumors. After intravenous injection, Th-TPZ@MOF-FA accumulated at the tumor site, releasing thrombin to activate platelets and induce vascular infarction, thereby increasing the hypoxic environment and activating TPZ. Both *in vivo* and *in vitro* investigations revealed that the Th-TPZ@MOF-FA nanosystems significantly amplified their anticancer potency, realizing a spontaneous synergistic treatment through vascular infarction and hypoxia-activated prodrugs. Similar studies were conducted by Zhao *et al.* 85, who utilized GEMT to exacerbate thrombosis, and by Zhu *et al.* 86, who employed Fe-TPZ to cause vascular damage through high temperatures.

Phototherapy (PDT and PTT), integrates real-time diagnosis with in-situ treatment, offering advantages such as non-invasiveness and low side effects. This makes it as a promising anti-tumor method 87. However, the efficacy of PTT or PDT when employed individually is often limited. To overcome this limitation, researchers have combined phototherapy with hypoxia-activated prodrugs to achieve a synergistic effect, thereby enhancing the efficacy of antitumor therapy 88. Zhang *et al*. 89 prepared nanogels, termed SiPNGs, by combining a “soft” polymer with “hard” silicon nanodots (SiND). These nanogels further encapsulated the photosensitizer precursors 5-ALA and TPZ to form 5-ALA/TPZ@SiPNGs. The nanogels were pH-responsive and released 5-ALA and TPZ in slightly acidic environment. 5-ALA was converted to the red-fluorescent PpIX, a process that concurrently consumed oxygen and generated singlet oxygen, thereby exacerbating hypoxia at the tumor site and activating the hypoxia-responsive cytotoxicity of TPZ. By leveraging the differential fluorescence intensity ratios between the red emission of PpIX and the green luminescence of SiPNGs, it was possible to distinguish cancer cells from normal cells. The 5-ALA/TPZ@SiPNGs represents the first cancer treatment platform to integrate ratiometric imaging, intelligent drug release, PDT and hypoxia-activated chemotherapy in a single system.

Cheng *et al.* 90 combined PCPDTBT and PS-TK-PEG to develop the nano-prodrug SPNST, in which a transfer inhibitor (SIS3) was introduced, thus further expanding the combination of phototherapy and hypoxia-activated prodrugs. Upon near-infrared laser irradiation, SPN_ST_ generated ^1^O_2_ to eliminate tumor cells, triggering the release of SIS3 and TPZ. SIS3 reduced tumor invasiveness by inhibiting the Smad3 signaling pathway, enabling SPN_ST_ to effectively target hypoxic tumors and inhibit tumor metastasis.

Leveraging the unique pH characteristics of the tumor microenvironment, Ihsanullah *et al*. 91 developed ^TAT+Azo^NPs composed of PEG-Azo-PLGA and ^DA^TAT-PEG-PLGA for the delivery of Ce6 and TPZ to the tumor region. Under acidic conditions, the amide bond between the TAT peptide and DA was cleaved, leading to the preferential degradation of DA protectants and the exposure of TAT peptides, thereby enhancing the penetration of ^TAT+Azo^NPs at the tumor site. This process improved PDT-induced distal tumor therapy and further induced hypoxia. The resulting hypoxic environment triggered the cleavage of the azobenzene bonds, leading to the degradation of ^TAT+Azo^NPs and the subsequent release of TPZ. This cascade of events generated cytotoxic free radicals that enhanced the therapeutic effect. The sequential activation of ^TAT+Azo^NPs@(Ce6+TPZ) in both proximal and distal tumor regions significantly improved the overall efficacy of therapy.

In addition, using pH-activated TAT peptides, Zhang *et al*. 92 developed a nanoplatform, ^DA^TAT-NP_VT_, which was capable of combining X-ray-induced photodynamic therapy (X-PDT) with hypoxia-responsive chemotherapeutic agents. In the acidic tumor microenvironment, 2,3-dimethylmaleic anhydride (DMMA) degraded, thereby reactivating the targeting function of the TAT peptides and enhancing the cellular internalization of ^DA^TAT-NP_VT_. Upon X-ray irradiation, encapsulated verteporfin (VP) underwent the X-PDT process, and the hypoxia induced by X-PDT further stimulated the release and activation of TPZ, producing pronounced anti-tumor effects. The same research group 93 also constructed a pH/hypoxia-responsive polymeric micelle, ^DA^NP_CT_, which incorporated Ce6 for its photodynamic properties and TPZ as a prodrug activated under hypoxic conditions. Utilizing a 660 nm laser, which exacerbated hypoxic conditions, thereby facilitating the dissociation of ^DA^NP_CT_ and activating the antitumor activity of TPZ. The synergistic effect significantly enhanced cytotoxicity to cancer cells.

Also using the combination of TPZ and Ce6 to enhance the therapeutic effect, Wu *et al*. 94 designed an active targeted drug delivery system, TPZ@HA-Ce6. TPZ was encapsulated in the hydrophobic cores of HA-Ce6 nanoparticles by ultrasonication. The HA component provided targeting specificity, allowing the nanoparticles to selectively target cancer cells that overexpress CD44 receptors through receptor-mediated endocytosis. Upon laser irradiation, Ce6 consumed oxygen, leading to the generation of highly efficient PDT and the subsequent activation of TPZ. The combined action of ROS produced by PDT and activated TPZ species in TPZ@HA-Ce6 nanomicelles results in favorable therapeutic effects.

Building on the application of HA for targeted therapy, Cheng *et al*. 95 loaded indocyanine green (IR820) and TPZ into mesoporous organic silica nanoparticles that can be decomposed by GSH. They prepared HA-TPZ&IR@GMON using HA as a linking agent. The nanomaterials degraded upon exposure to HAase and GSH, thereby releasing IR820 and TPZ. Under NIR irradiation, IR820 generated singlet oxygen, leading to increased tumor hypoxia and enhancing the chemotherapeutic effect of TPZ. The results showed that the nanomaterials prepared by this method could effectively inhibit tumor growth while exhibiting good biocompatibility, safety, and degradability. Zeng* et al.* 96 also conducted similar research utilizing the active targeting effect of folic acid, and PEI-FA@IR showed significant accumulation at the tumor site.

The specific receptor recognition capability of the iRGD peptide also enables targeted drug delivery to tumors. To overcome the challenges posed by the blood-brain barrier (BBB) and blood-brain-tumor barrier (BBTB) in glioma, while also activating hypoxia-responsive prodrugs, Zhang* et al*. 97 developed a liposome-based nanomedicine utilizing iRGD-mediated receptor targeting. Zinc phthalocyanine (ZnPc) and TPZ were encapsulated within liposomes and modified with the penetrating peptide iRGD to create iRGD@ZnPc+TPZ. The iRGD@ZnPc+TPZ overcame the blood-brain barrier and hypoxia through the integration of targeted modification and PDT.

**Figure 9:**
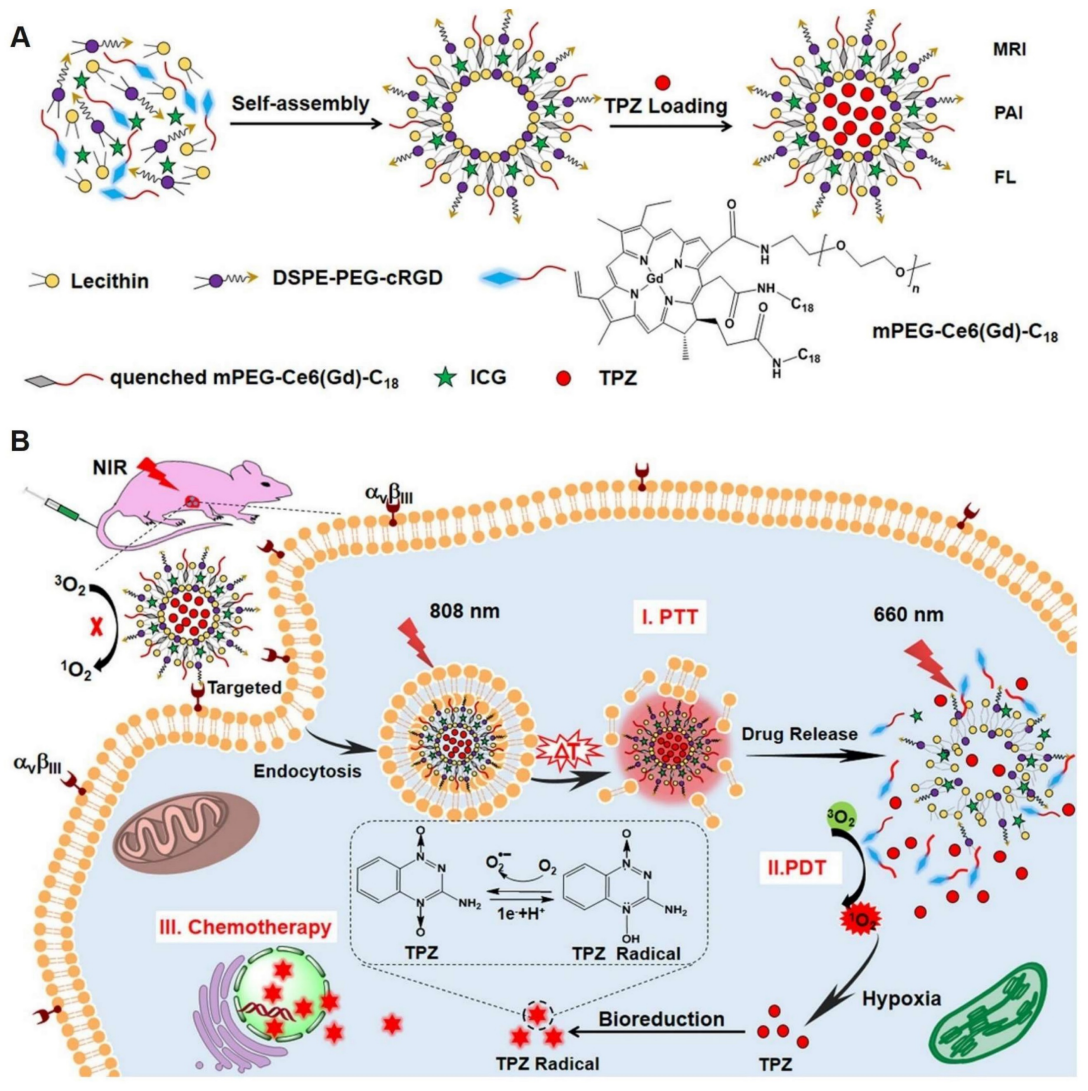
(A) The self-assembly synthesis process of ITC-Gd^III^ TLs. (B) Schematic diagram of NIR laser-triggered PDT, PTT and drug release. Adapted with permission from 98, copyright 2019, American Chemical Society.

Similar to iRGD, cRGD also facilitates deep penetration into tumors. Dai *et al*. 98 encapsulated the photothermal agent indocyanine green (ICG) and TPZ in liposomes, which were subsequently modified with cRGD and coupled with Gd^III^ to form ICG/TPZ@Ce6-Gd^III^ (ITC-Gd^III^ TLs). The cRGD-mediated targeting strategy effectively enriched nanoparticles at the tumor site. Under 808 nm NIR laser irradiation, the photothermal effect of ICG disrupted the ITC-Gd^III^ TLs, thereby activating the fluorescence and photodynamic effects of Ce6 and releasing TPZ. The ITC-Gd^III^ TLs activated a cascade of photothermal therapy (PTT), PDT, and chemotherapy, enhancing the overall antitumor response (Figure 9).

The microporous structure of MOFs facilitates the diffusion of ROS, enhancing the efficacy of PDT compared to other nanosized photosensitizers. Capitalizing on this property, Jia *et al*. 99 constructed a hybrid MOF nanomedicine, PAMNPs@TPZ, with enhanced PDT efficiency and oxygen consumption capabilities. The incorporation of AuNPs and PEG-SH stabilized the nanomedicine against phosphate in the blood, while TPZ was rapidly released in response to elevated intracellular phosphate concentrations.

Considering that inorganic nanocarriers such as metal-organic framework nanomaterials are difficult to biodegrade, Li *et al*. 100 synthesized a photodegradable polymer, pCy, by connecting cypate with mPEG_2k_-NH_2_ via amide bonds. This polymer subsequently self-assembled into TPZ-coated nanoparticles, designated as TPZ@pCy, which were prepared for the treatment of metastatic breast cancer and exhibited degradability under NIR irradiation. *In vivo* studies demonstrated that TPZ@pCy effectively promoted tumor ablation and reduced lung metastasis in tumor-bearing mice.

The clinical application of PDT is sometimes limited by its shallow tissue penetration depth. In contrast, Cu_2-x_Se exhibits efficient absorption in the NIR-II region, indicating superior tissue penetration. Chen *et al.* 101 designed chitosan (CS)-based nanoparticles co-loaded with copper selenium quantum dots (Cu_2-x_Se QDs) and TPZ, termed CS/Cu_2-x_Se-TPZ NPs. Under NIR-II laser irradiation, Cu_2-x_Se QDs as photosensitizers consumed oxygen and generated ^1^O_2_; additionally, they could also act as Fenton-like agents to convert H_2_O_2_ into ·OH, thereby killing cancer cells. The resulting exacerbation of hypoxia within the tumor microenvironment further promoted the activation of TPZ.

Compared to light-responsive nanoparticles, ultrasound (US) can penetrate tissue to a depth of 10 cm, making sonodynamic therapy (SDT) play a significant role in cancer treatment 102. Ding *et al.* 103 developed SPN_Ti_ nanoparticles that consume oxygen to produce ^1^O_2_ under sonic activation, resulting in the release of TPZ conjugates and ibrutinib. This strategy not only activated TPZ under hypoxic conditions but also reduced the immunosuppressive function of myeloid-derived suppressor cells, enhancing antitumor immunity. Additionally, Sheng *et al.* 104 incorporated perfluoropropane (PFP) into nanoparticles (PTP@PLGA) for sonodynamic therapy to relax the extracellular matrix and increase drug penetration at tumor sites, thereby further enhancing the therapeutic efficacy of TPZ combined with SDT.

**Figure 10:**
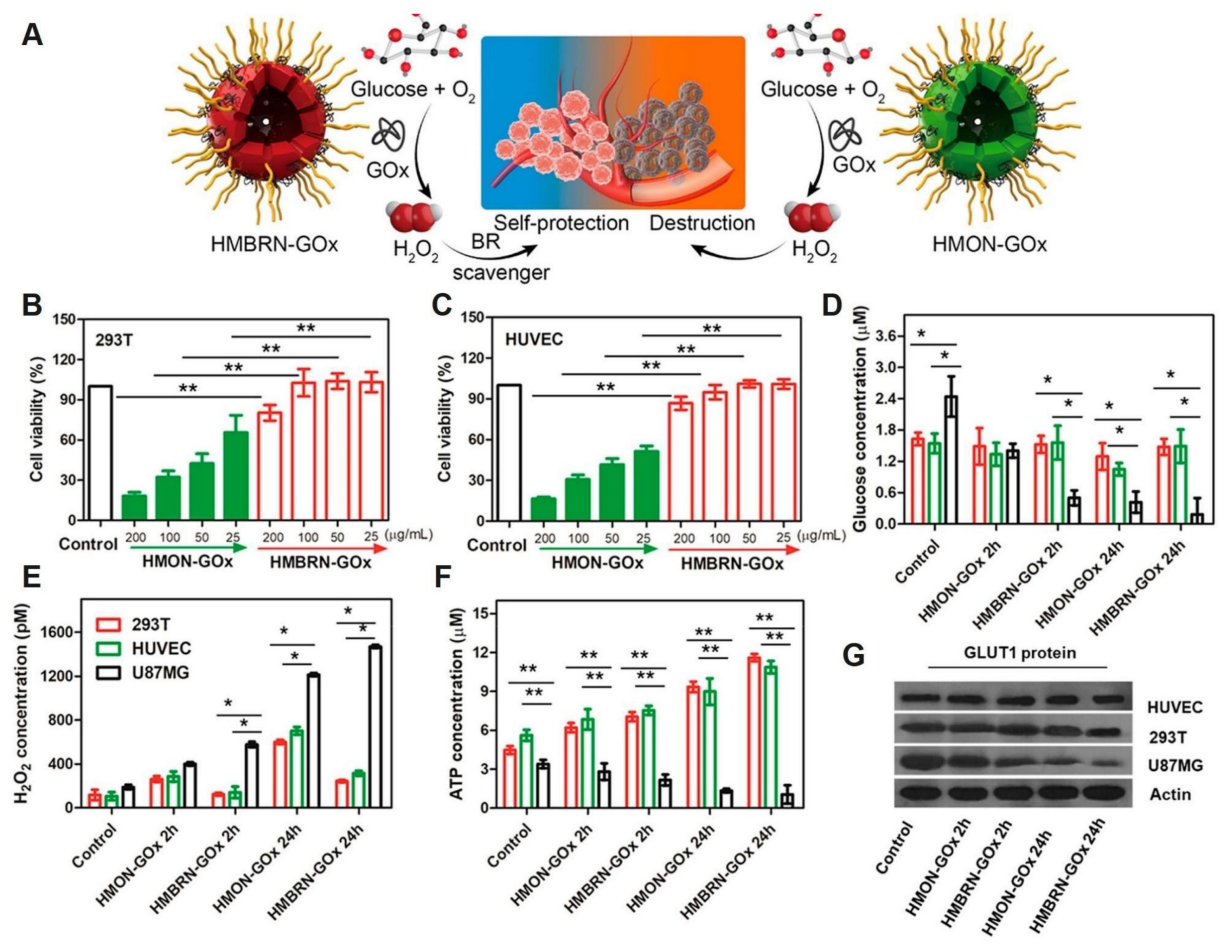
(A) Schematic of the opposite effects of HMON-GOx and HMBRN-GOx on normal tissues. Cytotoxicity of HMON-GOx and HMBRN-GOx on 293T cells (B) and HUVEC cells. (C) Concentrations of glucose (D), H_2_O_2_ (E), ATP (F) and GLUT1 protein expression (G) in 293T/HUVEC/U87MG cells after different treatment for 2h and 24 h, respectively. Adapted with permission from 106, copyright 2019, American Chemical Society.

In addition to the synergistic antitumor effects achieved by combining TPZ with phototherapy, researchers have also integrated TPZ with starvation therapy 105. Shan *et al*. 106 combined the principles of starvation therapy and chemotherapy to develop an organosilicon system, HMBRN-GOx/TPZ. Silicone-based hollow porous bilirubin nanoparticles (HMBRN) were capable of co-delivering glucose oxidase (GOx) and TPZ. This system protected normal tissues from oxidative damage by removing excess H_2_O_2_. It aggravated the hypoxic state of the tumor by rapidly consuming glucose and oxygen, thus improving the cytotoxicity of TPZ and effectively inhibiting the growth of solid tumors. Compared with traditional cancer starvation therapy, this research introduced a novel antioxidant-activated self-protective nanotechnology, significantly enhancing tumor-targeted hypoxia-based therapeutic effects. Importantly, this method does not require external energy, allowing for accurate endogenous cancer treatment with minimal side effects (Figure 10). Zhou *et al.* 107 also conducted similar work and realized the synergistic effect of TPZ and starvation therapy through PGT.

Employing a comparable strategy, Guo *et al*. 108 developed a self-amplifying nanoreactor, HGTFT, designed for a sustainable cascade of CDT that activates starvation and hypoxia responses. This system incorporated HSA, tannic acid (TA), GOx, Fe^3+^ and TPZ. The nanoreactor catalyzed the conversion of oxygen into ·OH for CDT, facilitated tumor starvation through glucose consumption, created an anoxic environment for TPZ-mediated chemotherapy, and released metal ions for metal ion interference therapy. The increased level of hypoxia further enhanced the conversion of TPZ into highly toxic radicals, thereby effectively inhibiting tumor growth. The tunable nature of HGTFT in the tumor microenvironment provides a sustainable cascade of antitumor effects.

Wang *et al*. 109 developed a nanomedicine based on the Janus nanoplatform, designated as FA-GT-MSNs@TPZ. FA-GT-MSNs@TPZ produced radiosensitization and photothermal toxicity, effectively killing normoxic tumor cells. Subsequently, TPZ was released, exerting cytotoxic effects on hypoxic tumor cells and enhancing the efficacy of radio-photothermal therapy. By integrating radiosensitization, photothermal therapy, and chemotherapy, the nanomedicine achieved a multi-modal therapeutic approach for liver cancer, demonstrating remarkable anti-tumor efficacy and excellent biosafety both *in vivo* and *in vitro*. FA-GT-MSNs@TPZ represents a promising strategy for the safe and effective treatment of liver cancer.

The multifunctional hydrogel Bi_2_S_3_/ALG@TPZ, constructed by Luo *et al.* 110, and the LCT, developed by Dong *et al.* 111, both employed a variety of synergistic therapeutic methods to achieve the clearance of tumor cells. Similar work was also conducted by Li *et al.* 112 in the design and synthesis of Ag_2_S@MSN-TGF. This nanoparticle utilized the enhanced penetration of PTT mediated by NIR-II laser and combined the synergistic method of starvation therapy, hypoxia-activated prodrug therapy, and PTT to enhance anti-cancer efficacy.

In recent years, it has been shown that photodynamic therapy can effectively induce immunogenic cell death (ICD) 113, 114. Wang *et al*. 115 developed an albumin-based multifunctional nanoplatform, IR780-NLG919-TPZ NPs, specifically designed to overcome the impediments to anti-tumor immunity posed by the immunosuppressive tumor microenvironment (TME) while simultaneously inducing ICD. Upon NIR irradiation, the photosensitizer IR780 produced ^1^O_2_ and concurrently cleaved the ROS-sensitive TK, triggering the release of TPZ. Under hypoxic conditions, TPZ was activated, contributing to the induction of ICD. The high levels of GSH present in the TME promoted the cleavage and activation of NLG919, thereby enhancing the infiltration of cytotoxic T lymphocytes (CTLs) within the tumor. The IR780-NLG919-TPZ NPs effectively addressed the challenges of tumor hypoxia, insufficient ICD induction, and immunosuppression. This nanoplatform provides a theoretical foundation for the treatment of hypoxic and immunosuppressed malignant tumors.

Ferroptosis, a novel cancer treatment method, has attracted significant attention. Currently, scientists are combining it with hypoxia-activated prodrugs to mitigate the chemical resistance associated with oxygen depletion during ferroptosis. Guo *et al.* 116 created Lip@PDA-Fe-TPZ nanoparticles by encapsulating iron ions and TPZ within PDA and modifying them with liposomes. These nanoparticles increased intracellular H_2_O_2_ levels and used iron to generate ·OH, thereby triggering ferroptosis and enhancing chemotherapeutic effect of TPZ under hypoxic conditions.

### Nanoparticles based on aliphatic nitrogen oxides

Similar to hypoxia-activated prodrug TPZ, Banoxantrone (AQ4N) can also be used in combination with photosensitizers to mitigate the negative effects of hypoxia on PDT. AQ4N, featuring a nitrogen-oxide quinone structure, is an effective and stable bioreductive prodrug 117. In its native state, AQ4N is largely non-toxic; however, it produces cytotoxicity following a two-step enzymatic reduction to AQ4M within hypoxic cells, which is then further converted into the topoisomerase II inhibitor AQ4 118, 119. Studies have shown that AQ4N can be effectively combined with chemotherapeutic agents or radiotherapy to augment its antitumor efficacy, with minimal or no increase in myelotoxicity 120. By designing intelligent nanomaterials, scientists have realized synergistic tumor treatments that combine PDT with hypoxia-activated chemotherapy, as detailed in this section (Table 5).

He *et al*. 121 reported a smart nanosystem based on UiO-66 NPs. These UiO-66 NPs were co-anchored with the photosensitizer photochlor (HPPH) and azide moieties, acting as nanocarriers for the bioreductive prodrug AQ4N, which was further modified by polyethylene glycol to obtain the A@UiO-66-H-P NPs. The AQ4N in these NPs was designed to be sensitive to phosphate ions, thereby minimizing premature leakage due to the differences in phosphate concentration between plasma or extracellular fluid and intracellular fluid during delivery. The hypoxia caused by PDT facilitated the conversion of AQ4N into its active form, thereby achieving the synergistic treatment of PDT and hypoxia-activated chemotherapy.

Photosensitizers can also serve as functional units for building organic frameworks, thereby enhancing the efficacy of PDT. Capitalizing on this concept, He *et al*. 122 combined the photosensitizer tetra(4-hydroxyphenyl)porphine (THPP) with a cleavable thioketal (TK) linker to form a COF nanoplatform with high porphyrin loading capacity, which significantly improved ROS production efficiency. The prepared nanoplatform was coated with PEG, culminating in the formation of AQ4N@THPPTK-PEG NPs. Upon 660 nm laser irradiation, porphyrin generated ^1^O_2_ through PDT while leaving the TK linker. This process resulted in the dissociation of the COF structure and the subsequent release of AQ4N. Zhang *et al.* 123 further coated the organic framework with PDA to synthesize APP NPs, thereby enabling simultaneous generation of PDT and PTT.

Photosensitizers not only exacerbate hypoxia but also indirectly induce ferroptosis, thereby enhancing the therapeutic efficacy of drugs. Luo *et al.* 124 encapsulated AQ4N, the photosensitizer iridium complex (Ir1), and the hepatocellular carcinoma-targeted therapy drug sorafenib within liposomes to form the AQ4N-Ir1-sorafenib-liposome. Upon light irradiation, Ir1 consumed O_2_ to produce ROS, resulting in the destruction of liposomes. Subsequently, sorafenib was released and AQ4N was activated. The released ^1^O_2_ oxidized GSH, inactivated GPX4, and induced the accumulation of lipid peroxides, thereby triggering ferroptosis. The AQ4N-Ir1-sorafenib-liposome offers a novel strategy for enhancing therapeutic efficacy in hepatocellular carcinoma.

Nanoparticles with adjustable sizes and charges can significantly reduce the adverse effects of drugs on healthy tissues. Ji *et al*. 125 created a nanoparticle with a tandem active mode of PDT and chemotherapy. This system, designated as (UCNP@PFNS/AQ4N)@MnCaP, consists of a NIR light-activated nanophotosensitizer (UCNP@PFNS) and AQ4N, coated with the pH-sensitive MnCaP. The encapsulation by MnCaP increased the size of the NPs and prolonged their circulation time. Upon reaching the tumor, the acidic conditions within the tumor microenvironment induced the disintegration of MnCaP, which facilitated the release of the smaller UCNP@PFNS and AQ4N, thereby enhancing drug penetration. Additionally, Mn^2+^ significantly amplified the MR signal at the tumor location. Under NIR light irradiation, UCNP@PFNS and AQ4N were released and successfully metastasized to the desired tumor area to exert potent cytotoxic effects, thereby enhancing the therapeutic outcome.

In recent years, scientists have also developed a number of nanocarriers based on cucurbit[7]uril (CB^7^) to enhance the delivery of AQ4N. Chen *et al*. 126 synthesized a derivative of CB^7^ functionalized with an AIE photosensitizer, designated as AIECB^7^. This derivative had the propensity to spontaneously aggregate into nanostructures in aqueous media. AQ4N was effectively incorporated into the AIECB^7^ nanostructures through host-guest interactions. Under weakly acidic conditions, AIECB^7^ was capable of generating singlet oxygen, a property that can be harnessed for synergistic chemo-photodynamic therapy.

Huang *et al*. 127 developed a supramolecular micelle based on PEG, which incorporates hydrophilic CB^7^ and hydrophobic Ce6. Folate-amantadine (FA-ADA) and AQ4N combined with CB^7^ through host-guest interactions to form AQ4N@CPC-FA. This complex utilized FA to selectively target tumors and was internalized by cancer cells to release AQ4N. Upon laser irradiation, Ce6 generated ^1^O_2_ for PDT, while simultaneously enhancing the activation of the chemotherapeutic drug AQ4N. In a similar study, Zhou *et al.* 128 incorporated FA into the design to synthesize AGPF NPs, which also successfully achieved active targeting of cancer cells.

Also using CB^7^ to optimize materials, Ding *et al*. 129 developed a new supramolecular nanomedicine platform, NGO-CB^7^, which combines CB^7^ with nano-graphene oxide (NGO). Through non-covalent π-π stacking and host-guest interactions, the platform successfully loaded Ce6, AQ4N, and oxaliplatin. The complex was then coated with ADA-tagged hyaluronic acid (ADA-HA) to form ACNGH^OX^. ACNGH^OX^ encapsulated OX through host-guest interactions, enabling surface functionalization and the incorporation of non-aromatic molecules. By leveraging the synergistic effects of photothermal and photodynamic therapies from NGO/Ce6, along with the dual chemotherapy regimen of OX and AQ4N, ACNGH^OX^ demonstrated significant antitumor effects.

## Conclusions and outlooks

In conclusion, this paper reviewed the application of hypoxia-responsive nanoparticles in cancer diagnosis and therapy. These nanoparticles, based on fluorescence imaging, can co-deliver fluorescent markers and chemotherapy drugs, enabling real-time monitoring of the drug release process. Additionally, they can encapsulate a variety of imaging agents to provide more information about tumor sites through multimodal imaging, thus coordinating tumor treatment and prognosis. Photosensitizers and photothermal agents produce singlet oxygen and heat, starvation therapy consumes glucose and oxygen or other ways in combination with chemotherapy to kill tumor cells and improve the efficacy of hypoxia-activated prodrugs. This integrated strategy for diagnosis and treatment not only increases the specificity of treatment, but also provides a possibility for reducing the side effects associated with chemotherapy.

With the rapid development of nanotechnology in fluorescence imaging and multi-modal therapy, its role in cancer diagnosis and treatment is becoming increasingly critical. But we should also clearly recognize that this model also has some shortcomings. Nanoparticles are readily captured by the reticuloendothelial system, leading to prolonged clearance periods, which pose potential residual risks. Therefore, the pharmacokinetic properties of nanoparticles *in vivo* should be thoroughly studied, and nanoparticles designed for rapid renal clearance should be developed to minimize residual accumulation in the body. In addition, fluorescence imaging primarily focuses on the visible light and the NIR-I regions, which are limited by the strong absorption and scattering properties of biological tissues, leading to suboptimal imaging depth and resolution. Although photoacoustic imaging and magnetic resonance imaging can make up for the shortcomings of fluorescence imaging, research on multi-modal imaging nanoparticles is still relatively scarce, and single imaging nanoparticles often struggle to provide accurate tumor diagnosis. Therefore, new nanoparticles with multiple imaging modalities need to be further developed to realize the complementary advantages between different imaging techniques and improve the accuracy of cancer diagnosis. Additionally, while nanoparticles guided by imaging technology for multimodal synergistic therapy have been widely studied, most studies are limited to cellular and animal models. Given the significant differences between human and animal models, it is necessary to explore the *in vivo* release mechanisms of nanoparticles and conduct more extensive clinical trials to evaluate their feasibility for clinical application. Although the EPR effect can passively target nanoparticles to tumor sites, its targeting is occasionally imprecise. Therefore, reasonable modifications to nanoparticles are necessary to enhance receptor-mediated endocytosis, thereby increasing the specific uptake of nanoparticles by tumor cells, which is a key way to improve therapeutic efficacy. Concurrently, the development of simpler, more cost-effective, and more intelligent nanomedicines represents a significant direction for future research.

Although nanoparticles have not yet been widely applied in clinical treatment, nanotechnology has shown remarkable potential in cancer diagnosis and therapy. With the continuous development of nanotechnology, it is expected that this technology will enable more accurate, efficient, and safe cancer diagnosis and treatment in the future. We hope that this review will serve as a detailed and comprehensive theoretical reference for researchers engaged in the design of nanoparticles.

## Figures and Tables

**Table 1:** Hypoxia-responsive nanoparticles based on fluorescence imaging.

NPs	Name	Type	Payloads	Size (nm)	*In vitro*/*in vivo* model	Imaging modality	Refs
1	FA-lip@NIR-NM	Liposome nanoparticles	NIR-NM	170.3	HepG-2/HeLa cells/LO2 cells/Female nude BABL/c mice	Fluorescence imaging	38
2	PCL_3k_-TPE-Azo-PEG_5k_	Polymer nanoparticles	DOX	—	Caco2 human colon cancer cells	Fluorescence imaging	39
3	mPEG_2k_-ADP-Azo@DOX	Micelles	DOX	40	L929/CT26 cells	Fluorescence imaging	40
4	Zr-MOF@PPa/AF@PEG	Metal-Organic Frameworks	AF/Ppa	87	HepG2/4T1 cells/Female BABL/c mice	Fluorescence imaging	41
5	PPEGMA_14_-ADP-Azo-PBzMAx	Micelles	DOX	33-162	HUVECs/CT26 cells	Fluorescence imaging	42
6	Cy7-NO_2_/HPC/TPZ NPs	Organic-inorganic hybrid nanoparticles	HPC/TPZ	96.7(1:5) 178.3(1:2)	CT26 cells/CT26 tumor-bearing BALB/c mice	Fluorescence imaging	43
7	Pep/BDP-NO_2_@Lip	Liposome nanoparticles	—	133	H9c2 cells/myocardial ischemic mice	Fluorescence imaging	44
8	HOISNDs	Metal nanoparticles	SPIONs	82	HepG2 cells/HepG2 tumor-bearing mice	Fluorescence imaging/MR imaging	45
9	UIO-Pimo	Metal nanoparticles	—	100	MDA-MB-231 cells/ BABL/c mice	Fluorescence imaging/MR imaging	46
10	M1-MPNPs	Biomimetic nanoparticles	PTX-NB	126	RAW264.7/4T1/MCF-10A/HK-2 cells/Female BALB/c mice	Fluorescence imaging/PA imaging	47
11	PCM@Lip/IT NPs	Liposome nanoparticles	TPZ/IR780	70	4T1 cells/Female nude BABL/c mice	Fluorescence imaging/PA imaging	48
12	CH-4T/SLB-MSN-MDOT/^64^Cu^2+^ (non-hypoxia-responsive)	Silicon-based nanoparticles	CH-4T	111.8 ± 0.2	A431/NIH 3T3 cells/ Nu/nu mice	Fluorescence imaging/PET imaging	49
13	Au@MSNs-ICG (non-hypoxia-responsive)	Silicon-based nanoparticles	ICG	90	HepG2-Fluc cells/BALB/c nu/nu male mice	Fluorescence imaging/CT imaging	50

**Table 2:** Samples of nitro-based nanoparticles.

NPs	Name	Type	Payloads	Size (nm)	*In vitro/in vivo* model	Therapeutic methods	Refs
1	TNP@DOX	Polymer nanoparticles	DOX	31.4	HeLa/HEK 293T cells	Chemotherapy	61
2	DOX-HR-LPs	Liposome nanoparticles	DOX	50	RM-1/FaDu cells/C57BL/6 mice/NOD/SCID mice	Chemotherapy	63
3	DOX-LPs	Liposome nanoparticles	DOX	50	RM-1/FaDu cells/C57BL/6 mice/NOD/SCID mice	Chemotherapy	63
4	PPGN@DOX	Polymer nanoparticles	DOX	72	4T1 cells/Female Balb/C mice	Chemotherapy	62
5	PPMs	Micelles	CPT/TPP	55±8	HeLa cells/BALB/c nude mice	Chemotherapy/PDT	64

**Table 3:** Samples of azobenzene-based nanoparticles.

NPs	Name	Type	Payloads	Size (nm)	*In vitro/in vivo* model	Therapeutic methods	Refs
1	HACB/BCNU NPs	Micelles	BCNU/*O*^6^-BG derivatives	190.47 ± 1.87	HeLa/A549/SMMC-7721/b End. 3 cells/ Female BALB/c nude mice	Chemotherapy	68
2	HCHOA	Biomimetic nanoparticle	Ce6/Oxaliplatin	100-150	4T1 cells/Female BALB/c nude mice	Chemotherapy/PDT	69
3	Glu-PEG-Azo-IR808-S-S-PTX	Polymer nanoparticles	PTX/IR808	50-100	A549 cell/A549 tumor-bearing nude mice	Chemotherapy/PTT	70
4	PAP NPs	Polymer nanoparticles	PTX	137.9 ± 3.5	HeLa/A549 cells/BALB/c mice	Chemotherapy	71
5	PCP_2K_ NPs	Polymer nanoparticles	PTX	160.1 ± 2.6	HeLa/A549 cells/BALB/c mice	Chemotherapy	71
6	Ce6@MSN/pDAB/F68	Silicon-based nanoparticles	DOX/Ce6	58.1 ± 6.0	MCF-7 cells/Female BALB/c mice	Chemotherapy/PDT	72
7	DOX@Biotin-SAC4A	Polymer Nanoparticles	DOX	223 ± 2	4T1/WI38 cells/ Female BALB/c mice	Chemotherapy	73

**Table 4:** Hypoxia-responsive nanoparticles based on benzotriazine nitrogen oxides.

NPs	Name	Type	Payloads	Size (nm)	*In vitro*/*in vivo* model	Therapeutic methods	Refs
1	GNPs-TPZ	Metal nanoparticles	TPZ	134 ± 75	MKN45 cells/Male nude mice	Chemotherapy	79
2	BCP-TPZ	Polymer nanoparticles	TPZ derivative	50	HeLa/HepG2 cells	Chemotherapy	80
3	TPZH-NPs	Polymer nanoparticles	TPZH	140	4T1 cells/Female BALB/c mice	Chemotherapy	81
4	PT-NPs	Polymer nanoparticles	TPZHex/PSM	113.9	4T1/HUVECs cells/Female BALB/c mice	Chemotherapy	82
5	TPZ/PFA@UiO-66@PDA	Metal-organic frame nanoparticles	TPZ/PFA	80-160	143B cells/male BALB/c nude mice	Chemotherapy/PTT	83
6	Th-TPZ@MOF-FA	Metal-organic frame nanoparticles	TPZ/thrombin	90	HepG2/COS7 cells/HepG2 tumor-bearing BALB/c nude mice	Chemotherapy/Vascular infarction	84
7	GEMT	Metal-organic frame nanoparticles	TPZ	181.16	L929/4T1 cells/BALB/c mice	Chemotherapy/MH	85
8	Fe-TPZ	Metal nanoparticles	Fe^2+^/TPZ	20	4T1/HUVECs/Hepa1-6/LO2 cells/C57 mice	Chemotherapy/RFA/ Immunotherapy	86
9	5-ALA/TPZ@SiPNGs	Silicon-based nanoparticles	5-ALA/TPZ	54.9±8.3	AT II/A549 cell/Female BALB/c nude mice	Chemotherapy/PDT	89
10	SPN_ST_	Polymer nanoparticles	TPZ/SIS3	22.2	4T1 cells/Female nude mice	Chemotherapy /PDT/Metastasis inhibition	90
11	^TAT+Azo^ NPs	Polymer nanoparticles	Ce6/TPZ	100	4T1 cells/Female BALB/c mice	Chemotherapy/PDT	91
12	^DA^TAT-NP_VT_	Polymer nanoparticles	VP/TPZ	80	MDA-MB-231 cells/Female BALB/c mice	Chemotherapy/X-PDT	92
13	^DA^NP_CT_	Polymer nanoparticles	TPZ/Ce6	80	MCF-7 cells/Female BALB/c mice	Chemotherapy/PDT	93
14	TPZ@HA-Ce6	Polymer nanoparticles	TPZ/Ce6	100	MDA-MB-231/4T1/ MCF-10A/ BALB/c mice	Chemotherapy/PDT	94
15	HA-TPZ&IR@GMON	Silicon-based nanoparticles	TPZ/IR820	100	4T1/LO2/L929 cells /Female BALB/c nude mice	Chemotherapy/PDT	95
16	PEI-FA@IR@TPZ/BMS LPs	Polymer nanoparticles	TPZ/IR820/BMS-202	81.5±4.2	4T1/A2780/bEND.3 cells/ Female BALB/c mice	Chemotherapy/PDT/ Immunotherapy	96
17	iRGD@ZnPc+TPZ	Liposome nanoparticles	TPZ/ZnPc	122	U87-MG/bEnd.3 cells/ BALB/c nude mouse	Chemotherapy/PDT	97
18	ITC-Gd^III^ TLs	Metal nanoparticles	ICG/TPZ	105 ± 5	A549/HeLa/NIH-3T3 cells/Female nude mice	Chemotherapy/PDT/PTT	98
19	PAMNPs@TPZ	Polymer nanoparticles	TPZ	65.3 ± 7.9	4T1 cells/BALB/c mice	Chemotherapy/PDT	99
20	TPZ@pCy	Micelles	TPZ	126	4T1 cells/BALB/c mice	Chemotherapy/PDT/PTT	100
21	CS/Cu_2-x_Se-TPZ NPs	Organic-inorganic hybrid nanoparticles	TPZ/Cu_2-*x*_Se QDs	167.25	4T1/MCF-10A/LX-2 cells/ Female BALB/c nude mice	Chemotherapy/PDT/CDT	101
22	SPN_ti_	Polymer nanoparticles	TPZ-conjugate/ ibrutinib	15.6±3.4	4T1 cells/Female BALB/c mice	Chemotherapy /SDT/Immunotherapy	103
23	PTP@PLGA	Polymer nanoparticles	TPZ/PFP/PpIX	302 ± 88.06	4T1 cells/Female BALB/c mice	Chemotherapy/SDT	104
24	HMBRN-GOx/TPZ	Silicon-based nanoparticles	TPZ/GOx	81.6 ± 3	U87MG/HUVEC/293T/MDA-MB-231 cells/Female BALB/c nude mice	Chemotherapy/ Starvation therapy	106
25	BP	Polymer nanoparticles	—	—	B16 cancer cells/C57BL/6 female mice.	PDT	107
26	HGTFT	Organic-inorganic hybrid nanoparticles	TPZ/GOx	24.8	4T1 cells/Female BALB/c mice	Chemotherapy/ Starvation therapy/Metal ion therapy	108
27	FA-GT-MSNs@TPZ	Silicon-based nanoparticles	TPZ	—	SMMC-7721/Hep3B/HL-7702/HUVEC Cells/male nude mice	Chemotherapy/RT/PTT	109
28	Bi_2_S_3_/ALG@TPZ	Organic-inorganic hybrid nanoparticles	TPZ	—	L929/mRBCs/HT29 cells/ tumor-bearing mice	Chemotherapy/RT/PTT	110
29	LCT	Liposome nanoparticles	CyI/TPZ	90	4T1 cells/BALB/c mice	PDT/PTT/Chemo/Immunotherapy	111
30	Ag_2_S@MSN-TGF	Silicon-based nanoparticles	TPZ/GOx	81	L929/HeLa cells/U14 tumor-bearing mice	Chemotherapy/ starvation therapy/PTT	112
31	IR780-NLG919-TPZ NPs	Biomimetic nanoparticle	IR780/TPZ	100-150	4T1 cells/Female BALB/c mice	Chemotherapy/PDT/Immunotherapy	115
32	Lip@PDA-Fe-TPZ	Liposome nanoparticles	TPZ/Fe	110	4T1/3T3 cells/Female BALB/c mice	Chemotherapy/Ferroptosis therapy	116

**Table 5:** Hypoxia-responsive based on aliphatic nitrogen oxides.

NPs	Name	Type	Payloads	Size (nm)	*In vitro*/*in vivo* model	Therapeutic methods	Refs
1	A@UiO-66-H-P NPs	Metal-organic frame nanoparticles	HPPH/AQ4N	95	U87MG cells/Female nude mice	Chemotherapy/PDT	121
2	AQ4N@THPP_TK_-PEG NPs	Covalent organic frame nanoparticles	THPP/AQ4N	67	4T1/Hela/L929 cells/ Female BALB/c mice	Chemotherapy/PDT	122
3	APP NPs	Metal-organic frame nanoparticles	AQ4N	105	HUVECs/4T1 cells/4T1 tumor-bearing mice	Chemotherapy/PDT/PTT	123
4	AQ4N-Ir1-sorafenib-liposome	Liposome nanoparticles	AQ4N/Ir1/sorafenib	150	HepG2 cells	Chemotherapy/PDT/Ferroptosis therapy	124
5	(UCNP@PFNS/AQ4N)@MnCaP	Organic-inorganic hybrid nanoparticles	UCNP@PFNS/AQ4N	73	HeLa cells/Male BALB/c mice	Chemotherapy/PDT	125
6	AIECB^7^	Organic nanoparticles	AQ4N/ Oxaliplatin	—	LO2/A549 cells	Chemotherapy/PDT	126
7	AQ4N@CPC-FA	Supramolecular micelles	Ce6/AQ4N	157.2	A12/MCF-7/4T1 cells/Male BALB/c nude mice	Chemotherapy/PDT	127
8	AGPF NPs	Organic-inorganic hybrid nanoparticles	AQ4N/Gd^3+^	141.8±6.8	U87 cells/SCID mice	Chemotherapy/PTT	128
9	ACNGH^OX^	Organic-inorganic hybrid nanoparticles	Ce6/AQ4N/OX	150	B16/LO2 cells/Female C57BL/6 mice	Chemotherapy/PDT/PTT	129
